# Selective Catalytic Synthesis of 1,2‐ and 8,9‐Cyclic Limonene Carbonates as Versatile Building Blocks for Novel Hydroxyurethanes

**DOI:** 10.1002/chem.201905561

**Published:** 2020-05-08

**Authors:** Katarzyna A. Maltby, Marc Hutchby, Pawel Plucinski, Matthew G. Davidson, Ulrich Hintermair

**Affiliations:** ^1^ Centre for Sustainable and Circular Technologies University of Bath Claverton Down BA2 7AY Bath UK

**Keywords:** biomass, renewable resources, epoxidation, polyoxometalates, polymers

## Abstract

The selective catalytic synthesis of limonene‐derived monofunctional cyclic carbonates and their subsequent functionalisation via thiol–ene addition and amine ring‐opening is reported. A phosphotungstate polyoxometalate catalyst used for limonene epoxidation in the 1,2‐position is shown to also be active in cyclic carbonate synthesis, allowing a two‐step, one‐pot synthesis without intermittent epoxide isolation. When used in conjunction with a classical halide catalyst, the polyoxometalate increased the rate of carbonation in a synergistic double‐activation of both substrates. The *cis* isomer is shown to be responsible for incomplete conversion and by‐product formation in commercial mixtures of 1,2‐limomene oxide. Carbonation of 8,9‐limonene epoxide furnished the 8,9‐limonene carbonate for the first time. Both cyclic carbonates underwent thiol–ene addition reactions to yield linked di‐monocarbonates, which can be used in linear non‐isocyanate polyurethanes synthesis, as shown by their facile ring‐opening with *N*‐hexylamine. Thus, the selective catalytic route to monofunctional limonene carbonates gives straightforward access to monomers for novel bio‐based polymers.

## Graphical abstract

**Biopolymers**: The selective catalytic synthesis of limonene‐derived monofunctional cyclic carbonates and their subsequent functionalisation via thiol–ene addition and amine ring‐opening is reported. The selective catalytic route to monofunctional limonene carbonates gives straightforward access to monomers for novel bio‐based polymers.

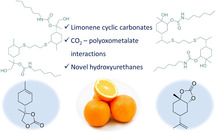

## Introduction

Terpenes are a large and diverse class of hydrocarbons naturally occurring in the oils of plants^1^ and fruits that have been used for flavour and fragrance manufacturing since antiquity.^2^ Due to their structural diversity and low oxygen content, investigations into their use as renewable feedstocks for chemical building blocks^3^, ^4^, ^5^ have recently received increasing attention.^6^ The most prevalent and widely available terpenes are limonene, α‐ and β‐pinene. Pinene is typically extracted from crude turpentine, a by‐product of the paper industry generated at a scale of 300 000 tonnes per annum, containing 40–85 % α‐pinene and 0.5–28 % β‐pinene.^7^, ^8^ Limonene is a by‐product of the citrus industry with an estimated global production of 70 000 tonnes per annum.^9^ The range and volume of available terpenes may be even higher through industrial biotechnology to provide geographically flexible supplies of terpenes via fermentation of plant sugars and cellulose waste.^10^, ^11^ In contrast to other renewable feedstocks such as carbohydrates, terpenes are partially unsaturated hydrocarbons that are chemically similar to petrochemical feedstocks long used in industry. Their potential to be used as drop‐in replacements for renewable commodity manufacturing through well‐established olefin chemistry makes terpenes particularly attractive biogenic feedstocks.^12^ However, in order to develop truly sustainable processes based on terpene feedstocks we will need sustainable and scalable methodologies tailored to their upgrading.^13^

**Figure 1:**
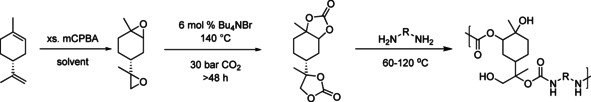
Limonene‐based NIPU synthesis via 1,2,8,9‐limonene cyclic carbonate according to Mülhaupt.^22^, ^23^

Limonene (**1**) and other terpenes have been used in the synthesis of a variety of building blocks,^14^, ^15^, ^16^, ^17^, ^18^, ^19^, ^20^ including cyclic carbonates,^21^, ^22^, ^23^ which have subsequently been used for the synthesis of non‐isocyanate polyurethanes (NIPU). NIPUs have similar properties to commercially available polyurethanes, but avoid the use of toxic isocyanates in the synthesis.^24^ Thus, the development of terpene‐based cyclic carbonates can lead to new bio‐based polymer materials with unexplored and potentially tuneable properties. This assumption is supported by recent work of Mülhaupt on 1,2,8,9‐limonene carbonate and related NIPU thermosets and thermoplastics (Figure 1).^22^, ^23^ The initially synthesised limonene‐derived NIPUs yielded rigid and brittle pre‐polymers,^22^ but subsequent work reported a modification that allowed for an improved purification of 1,2,8,9‐limonene carbonate that significantly reduced the colourization and improved the mechanical and thermal properties of the resulting limonene‐based NIPUs.^23^

Thus, clean, efficient and scalable routes to these cyclic carbonates are key to sustainable manufacturing of terpene‐based NIPUs. The quaternary ammonium salt used by Mülhaupt is a cheap and relatively efficient catalyst for the insertion of CO_2_ into the epoxide moieties of 1,2,8,9‐limonene bis‐oxide. A wide range of catalysts for the synthesis of cyclic carbonates from epoxides has been described in literature, including metal complexes, metal organic frameworks, quaternary ammonium salts, imidazolium halide salts and supported ionic liquid phase catalysts.^25^, ^26^, ^27^, ^28^, ^29^, ^30^, ^31^, ^32^ After formation of cyclic carbonates such as 1,2,8,9‐limonene carbonate, the ability to further modify the monomer or carry out post‐polymerization functionalisation is an important factor. With their multiple sites of unsaturation, terpenes can be ideally exploited in this way; however, examples such as 1,2,8,9‐limonene carbonate can only be tuned through the linking co‐monomer. Selectively functionalising only one of the double bonds available would provide further opportunities to introduce additional modifications.

Selective formation of 1,2‐limonene cyclic carbonate from commercially available 1,2‐limonene oxide (**2**) has previously been investigated (Table 1). All of the catalysts used showed more favourable formation of *trans*‐1,2‐limonene cyclic carbonate (*trans*‐**3**) over the *cis* isomer (*cis*‐**3**). Although some catalysts mediate the carbonation reaction at milder temperatures of 75–100 °C and lower pressures in the range of 10–50 bar, these reactions required long reaction times of 16–66 h. Moreover, some systems required the use of organic solvents (Table 1, entries 1 and 2) and co‐catalytic additives (entries 1–5). All of these studies were performed using commercially available **2**, and no system has thus far been investigated for performing an epoxidation–carbonation tandem reaction.

**Table 1:** Literature reports of the carbonation of commercial **2**.

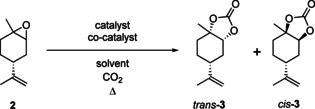									
Entry	Catalyst [mol %]	Co‐catalyst [mol %]	Solvent	*T* [°C]	*t* [h]	*p* [bar]	Yield **3** [%]	*cis* **‐3**/*trans* **‐3**	Ref.
1	CaI_2_ (10) crown ether derivative (10)	Ph_3_P (10)	MeCN	75	48	50	80	14:86	33
2	Al amino trisphenolate (1)	[PPN]Cl (3)	MEK	85	66	10	57	1:99	21
3	lanthanum heteroscorpionate (1)	[Bu_4_N]Cl (4)	neat	100	16	10	43	8:92	34
4	trimetallic cobalt complex (0.1)	[Bu_4_N]Cl (5)	neat	80	24	20	33	not reported	35
5	Al complex (1)	[Bu_4_N]Cl (3)	neat	80	66	10	48	8:92	36
6	formazanate ferrate(II) (1)	none	neat	90	18	12	2	not reported	37

**Figure 2:**
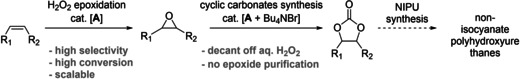
General scheme of epoxidation–carbonation sequence catalysed by **A**.

Recently, a solvent‐free protocol for limonene epoxidation was reported in which a catalyst consisting of an ammonium phase transfer catalyst (PTC) and the [PW_4_O_24_]^3−^ polyoxometalate anion ([PTC]_3_[PW_4_O_24_]=**A**) yielded 94 % 1,2‐limonene oxide after 1 h reaction time at room temperature using H_2_O_2_ as the oxidant.^38^ Although **A** is highly efficient for limonene epoxidation under these conditions, it was shown to be difficult to recover and lost activity upon recycling. Thus, instead of recycling the catalyst for repeat epoxidation runs, we set out to test whether it could be used in the following carbonation reaction without intermittent epoxide isolation (Figure 2).

**Figure 3:**
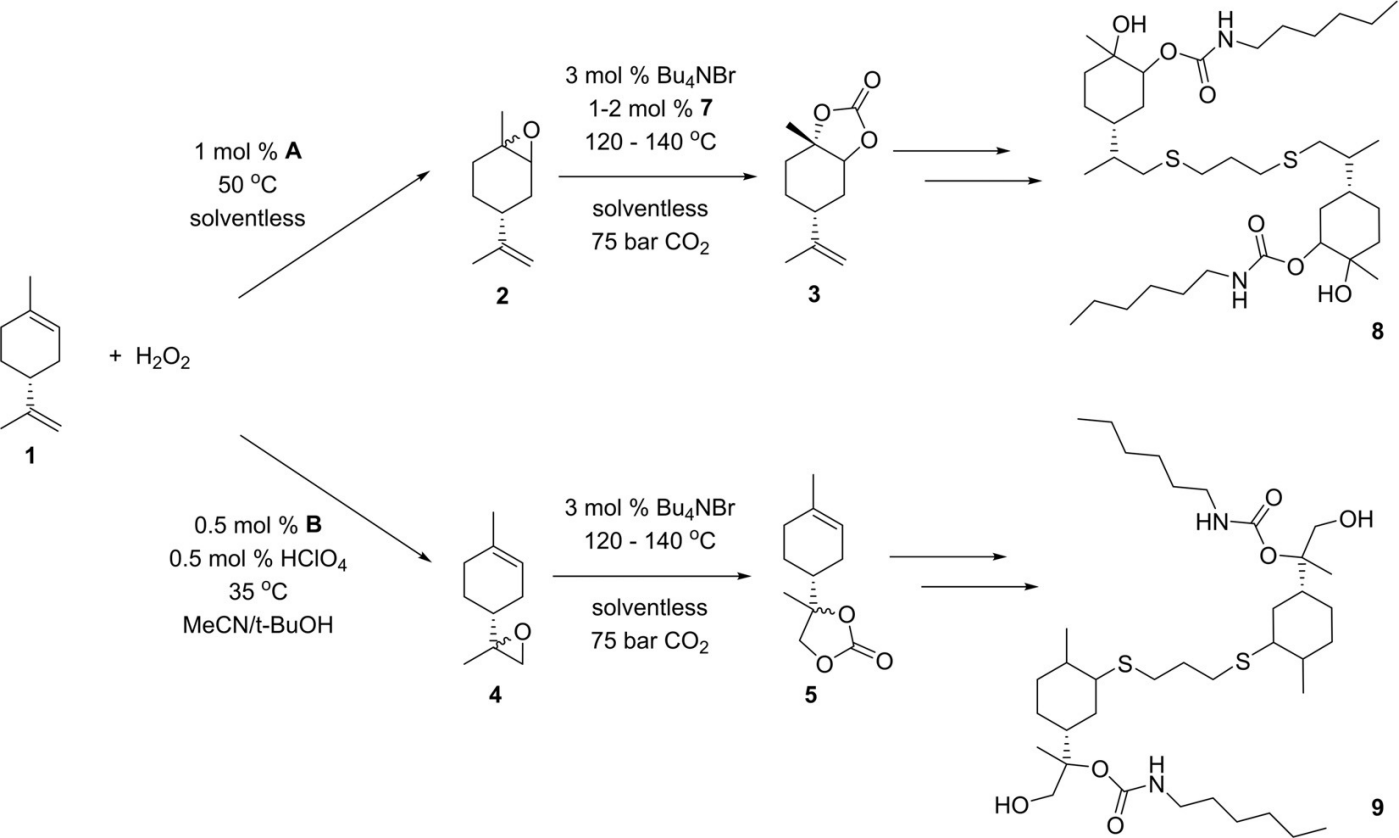
Limonene cyclic carbonates and corresponding hydroxyurethanes investigated in this work.

Here, we report the development of such an effective catalytic system that gives access to *trans*‐1,2‐limonene cyclic carbonate (**3**) and 8,9‐limonene cyclic carbonate (**5**) using benign oxidants and solvents (Figure 3). We found that for 1,2‐limonene oxide (**2**), a single polyoxometalate catalyst (**A**) can catalyse both the epoxidation and the CO_2_ insertion step. Furthermore, we demonstrate effective CO_2_ insertion to 8,9‐limonene oxide (**4**) for the first time. Both chiral cyclic carbonates are shown to undergo radical coupling with 1,3‐propanedithiol and react with *N*‐hexylamine to result in novel hydroxyurethanes as a demonstration of their utility in the synthesis of bio‐based NIPUs.

## Results and Discussion

Polyoxometalates such as those originally reported by Ishii and Venturello, [PTC]_3_[PW_4_O_24_] (PTC=methyl tricapryl ammonium) (**A**) are excellent catalytic systems for the epoxidation of a variety of alkenes, including terpenes.^39^, ^40^, ^41^, ^42^ Limonene epoxidation using **A** may take place under solvent‐free conditions and mild temperatures using H_2_O_2_ as an oxidant. Moreover, the reaction can be set up in batch or in flow reactor, reaction times are short (10 min residence time in a flow setup), and the selectivity toward epoxides is as high as 90 %.^38^ We therefore investigated the possibility of telescoping the benign epoxidation using **A** directly into the synthesis of cyclic carbonates. Silicotungstate polyoxometalates, such as [(*n*‐C_7_H_15_)_4_N]_6_[α‐SiW_11_O_39_Co] and [(*n*‐C_7_H_15_)_4_N]_6_[α‐SiW_11_O_39_Mn], have been reported as an efficient nonhalogen anionic catalyst for coupling CO_2_ and epoxides.^43^ Moreover, a combination of spectroscopic techniques and computational studies showed that transition‐metal polyoxometalates can coordinate CO_2_.^44^, ^45^ Leitner and co‐workers reported synergistic effects of [Bu_4_N]Br and [(*n*‐C_7_H_15_)_4_N]_5_[CrSiW_11_O_39_] in carbonation reactions of unsaturated carboxylic acids to give increased reaction rates and improved stereoselectivity.^46^ The prominent non‐silicotungstate epoxidation catalyst **A** has not yet been investigated for its ability to catalyse epoxide carbonation.

### Carbonation of cyclohexene oxide

We first investigated catalytic CO_2_ insertion into cyclohexene oxide (**10**) as a model compound under solvent‐free conditions. At 120 °C [Bu_4_N]Br showed good activity, providing 53 % of the corresponding cyclic carbonate **11** after 4 hours (Table 2, entry 3). The epoxidation catalyst **A** was found to be more active under the same conditions, however, with lower selectivity, giving about the same yield of **11** (Table 2, entry 2). To our delight we found that a combination of [Bu_4_N]Br and **A** gave high activity whilst maintaining high selectivity to give **11** in >80 % yield after 4 hours at 120 °C (Table 2, entry 1) through a synergistic double‐activation of both substrates, as in a related system reported by Leitner.^46^ Although **A** could not be recycled from these reactions either, the opportunity to use the same catalyst for two consecutive transformations represents a significant advantage over its single use in epoxidation only (see Table 8 below).

**Table 2:** Cyclohexene cyclic carbonate synthesis from cyclohexene oxide catalysed by **A** and [Bu_4_N]Br.^[a]^

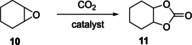						
Entry	Catalyst [mol %]	*T* [°C]	*p* [bar]^[b]^	*t* [h]	Conv. [%]^[c]^	Chemoselectivity **11** [%]^[c]^
1	**A** (2)+[Bu_4_N]Br (3)	120	75	4	97	84
2	**A** (2)	120	75	4	78	69
3	[Bu_4_N]Br (3)	120	75	4	60	88
4	none	120	75	4	0	–

[a] Reaction conditions: **10** (6.3 mmol), biphenyl (10 mol %) as an internal standard. [b] Pressure of CO_2_ at 40 °C. [c] Derived from quantitative ^1^H NMR spectroscopy.

### Carbonation of commercially available 1,2‐limonene oxide

Following these encouraging results, the same methodology was applied to commercially available **2** to evaluate the difference between CO_2_ insertion catalysed by [Bu_4_N]Br and the combined catalytic system consisting of [Bu_4_N]Br and **A** more closely (Table 3). Initially we applied 3 mol % [Bu_4_N]Br at 30 bar CO_2_, which gave high conversion but with poor selectivity (20 %), with higher pressures of 75 bar improving selectivity to 41 % (entry 2). The addition of 2 mol % **A** to 3 mol % [Bu_4_N]Br improved both conversion and selectivity further to give **3** in about 40 % yield after 24 hours (entry 3). An increased loading of [Bu_4_N]Br alone gave even higher conversion but chemoselectivity to **3** was still below 60 % (entry 4).

**Table 3:** 1,2‐Limonene carbonate synthesis from commercially available 1,2‐limonene oxide.^[a]^

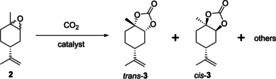						
Entry	Catalyst [mol %]	*T* [°C]	*p* [bar]^[b]^	Conv. [%]^[c]^	Chemoselectivity **3** [%]^[c]^	*trans*/ *cis* **3**
1	[Bu_4_N]Br (3)	140	30	79	20	96:4
2	[Bu_4_N]Br (3)	140	75	61	41	94:6
3	**A** (2)+[Bu_4_N]Br (3)	140	75	73	53	92:8
4	[Bu_4_N]Br (5)	140	75	95	52	95:5

[a] Reaction conditions: **2** (2.3 mmol), biphenyl (10 mol %) as an internal standard, 24 h, solventless. [b] Pressure of CO_2_ at 40 °C. [c] Determined by quantitative ^1^H NMR spectroscopy.

**Figure 4:**
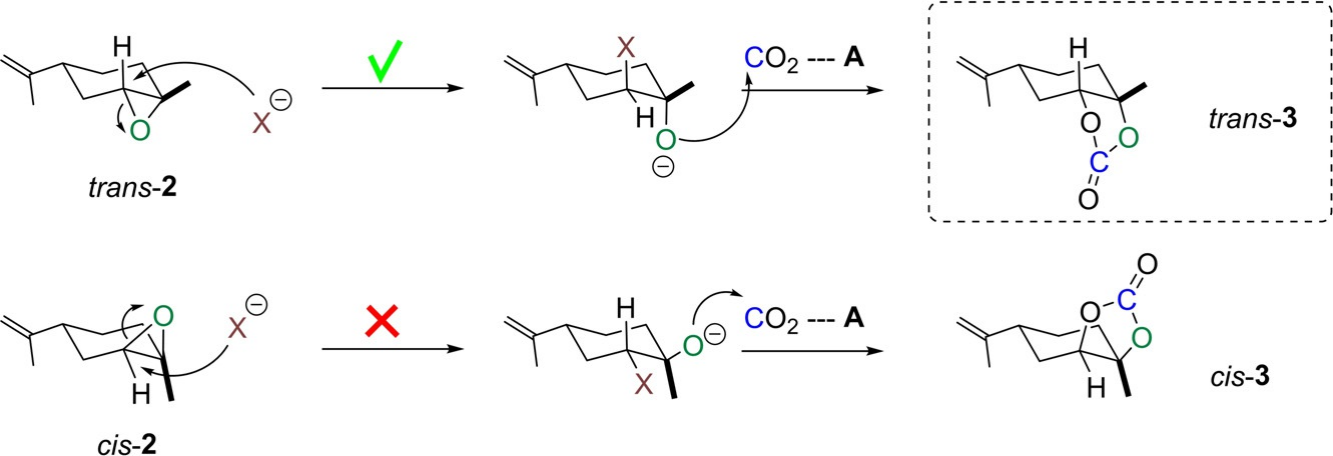
Different reactivity of *cis*/*trans* isomers of 1,2‐limonene oxide in the nucleophilic halide activation for CO_2_ insertion.^21^, ^46^

Interestingly, all catalysts and conditions tested predominantly produced the *trans* isomer of **3** with >90 % stereoselectivity. Starting with a 6:4 mixture of *trans*/*cis* epoxide isomers in commercial **2**, analysis of the ^1^H NMR spectra of the crude reaction mixture showed predominantly *cis*‐**2** to remain after the reaction. This finding suggested *cis*‐**2** and *trans*‐**2** differ in their reactivity towards CO_2_ insertion; an observation that has also been reported by Kleij and co‐workers recently.^21^ Indeed, considering conformational and steric effects of the double inversion pathway of halide‐catalysed CO_2_ insertion (Figure 4), it is clear that nucleophilic activation of *cis*‐**2** by halide (X^−^) is less favourable than in the case of the more easily accessible *trans*‐**2**.^21^, ^47^ Although we have not computed their thermodynamics, we expect the carbonate isomers *cis*‐**3** and *trans*‐**3** to be very close in energy, and thus propose the observed discrimination between *cis*‐**2** and *trans*‐**2** in the CO_2_ insertion step to be kinetic in nature.

### Reactivity of *cis*‐ and *trans*‐1,2‐limonene oxides in carbonation

**Figure 5:**
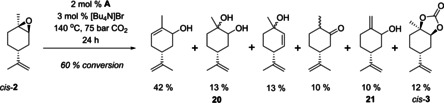
Product distribution from the attempted CO_2_ insertion into *cis*‐**2** as determined by GC‐MS and NMR spectroscopic analysis (% selectivity).

**Figure 6:**
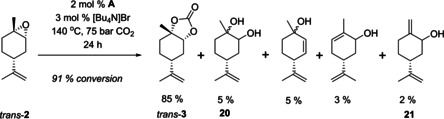
Product distribution from CO_2_ insertion into *trans*‐**2** as determined by GC‐MS and NMR spectroscopic analysis (% selectivity).

To investigate this assumption further, we separated the two isomers of **2** according to previously reported methods.^48^, ^49^ In line with our catalytic results using the commercial 6:4 mixture, the *cis* isomer of **2** alone did get converted but with very poor chemoselectivity to **3** (Table 4, entry 1). Instead, a number of hydrolysis and rearrangement products were obtained under the conditions applied (Figure 5). The pure *trans* isomer of **2** however showed good reactivity and excellent chemoselectivity to *trans*‐**3** under the same conditions (Table 4, entries 5 and 6, and Figure 6). Thus, not only is *trans*‐**2** clearly more reactive towards carbonation than *cis*‐**2**, but the latter gives rise to unwanted side products instead.

**Table 4:** Reactivity of different mixtures of *cis*‐**2** and *trans*‐**2** towards CO_2_ insertion.^[a]^

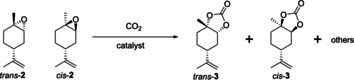						
Entry	*trans*/*cis* **2**	*t* [h]	Conv. [%]^[b]^	*trans*/*cis* **2** (after reaction)	Chemoselectivity **3** [%]	*trans*/*cis* **3**
1	0:100	24	60	0:100	12	0:100
2	13:87	24	80	13:87	26	55:45
3	57:43	24	83	33:67	64	99:1
4	97:3	24	91	91:9	85	99:1
5	97:3	4.5	60	96:3	95	98:2
6	100:0	4.5	61	100:0	89	100:0

[a] Reaction conditions: **2** (4.6 mmol), [Bu_4_N]Br (3 mol %), **A** (2 mol %), biphenyl (10 mol %) as an internal standard, 140 °C, 75 bar CO_2_ at 40 °C, solventless. [b] Determined from quantitative ^1^H NMR spectroscopy.

**Figure 7:**
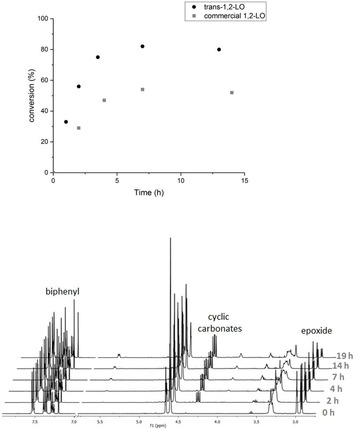
Top: Reaction profiles at 140 °C and 75 bar CO_2_, **2** (2.83 mmol), **A** (2 mol %), [Bu_4_N]Br (3 mol %), biphenyl (10 mol %) as an internal standard. Commercial **2** was sourced from Sigma–Aldrich, *trans*‐**2** has been synthesised in the procedure described in the Supporting Information, it is optically 100 % *trans*‐**2** containing 10 % impurities (see the Supporting Information IVc). Bottom: ^1^H NMR spectra showing reaction progress. The presented spectra correspond to data points collected for commercial **2** (57:43 *trans*/*cis*).

Reaction profiles of a commercial sample of **2** (57:43 *trans*/*cis*) showed global conversion of the mixture to be lower than for the pure *trans* isomer due to a lower concentration of the reactive isomer (Figure 7).

### Temperature influence on carbonation of *trans*‐1,2‐limonene oxide

Varying the reaction temperature from 40–140 °C with pure *trans*‐**2** showed that conversion strongly depended on temperature (Table 5). Applying 120 °C resulted in lower conversion than at 140 °C, but the reaction maintained high carbonate selectivity. Lowering the temperature further to 80 °C showed significantly diminished conversion and selectivity, and at 40 °C no reactivity was observed at all. These findings are consistent with data previously reported for 1,2,8,9‐limonene carbonate, in which the conversion was high for reactions between 120–140 °C but significantly lower at 100 °C.^22^ Reaction temperatures above 100 °C are typical for cyclic carbonate synthesis catalysed by quaternary ammonium salts; however, the optimum reaction temperature often depends strongly on the epoxide used.^43^, ^46^, ^50^, ^51^ For *trans*‐**2**, conversion increased with higher temperature and the high selectivity of the reaction was not affected up to 140 °C.

**Table 5:** The influence of temperature on *trans*‐**3** synthesis.

Entry	Catalyst	*T* [°C]	*t* [h]	Conv. [%]^[b]^	Selectivity *trans*‐**3** [%]	*trans*/*cis* **3**
1	**A**+[Bu_4_N]Br	140	4.5	65	80	1:0
2	**A**+[Bu_4_N]Br	120	4.5	42	78	1:0
3	**A**+[Bu_4_N]Br	80	4.5	10	45	1:0
4	**A**+[Bu_4_N]Br	40	4.5	5	0	–

[a] Reaction conditions: *trans*‐**2** (1.73 mmol) (see the Supporting Information IVc), **A** (2 mol %), [Bu_4_N]Br (3 mol %), biphenyl (10 mol %) as an internal standard, stirrer speed 300 rpm, 75 bar CO_2_ at 40 °C. [b] Determined from quantitative ^1^H NMR spectroscopy.

### Pressure influence on carbonation of *trans*‐1,2‐limonene oxide

Varying the CO_2_ pressure in the range of 20–75 bar (corresponding to CO_2_ densities of 0.037–0.238 g mL^−1^) showed that selectivity was strongly affected by the amount of CO_2_ added (Table 6). At CO_2_ densities between 0.15 and 0.24 g mL^−1^ (60–70 bar) conversion and selectivity remained high. However, reactions carried out at CO_2_ densities of 0.06 g mL^−1^ (30 bar) or below showed significantly lower selectivity whilst retaining the same level of substrate conversion. A reaction carried out under atmospheric pressure of air yielded mostly hydrolysis and rearrangement products **20** and **21** from non‐productive epoxide activation by the halide (Table 6, entry 5).

**Table 6:** The influence of pressure on *trans*‐**3** synthesis.^[a]^

Entry	Catalyst	*T* [°C]	*p* [bar]^[b]^	*t* [h]	Conv. [%]^[c]^	Selectivity *trans*‐**3** [%]	*trans*/*cis* **3**
1	**A**+[Bu_4_N]Br	140	75	4.5	68	76	1:0
2	**A**+[Bu_4_N]Br	140	60	4.5	62	79	1:0
3	**A**+[Bu_4_N]Br	140	30	4.5	65	55	1:0
4	**A**+[Bu_4_N]Br	140	20	4.5	45	35	1:0
5	**A**+[Bu_4_N]Br	140	1^[d]^	4.5	16	25^[e]^	1:0

[a] Reaction conditions: *trans*‐**2** (2.16 mmol) (see the Supporting Information IVc), **A** (2 mol %), [Bu_4_N]Br (3 mol %), biphenyl (10 mol %) as an internal standard, stirrer speed 300 rpm. [b] Pressure of CO_2_ at 40 °C. [c] Determined from quantitative ^1^H NMR spectroscopy. [d] Atmospheric pressure of air. [e] Low concentration of product; main side products identified as **20** and **21** by GC‐MS.

### Catalyst variation

When reducing the amount of **A** from 2 to 1 mol % (Table 7, entries 1–3) the conversion decreased by 12 % with no significant changes in selectivity. It was also found possible to use 5 mol % **A** without any addition of [Bu_4_N]Br (Table 7, entry 4), but at a rather unfavourable mass ratio (5 mol % **A** being equal to 80 weight% of the reaction).

**Table 7:** The synthesis of *trans*‐**3** with various catalysts.^[a]^

Entry	Catalyst [mol %]	*t* [h]	Conv. [%]^[b]^	Selectivity *trans*‐**3** [%]	*trans*/ *cis* **3**
1	**A** (2)+[Bu_4_N]Br (3)	4.5	68	76	1:0
2	**A** (1)+[Bu_4_N]Br (3)	4.5	56	77	1:0
3	**A** (1)+[Bu_4_N]Br (3)	24	91	64	1:0
4	**A** (5)	4.5	83	72	1:0
5	[Bu_4_N]Cl (3)	4.5	35	82	1:0
6	[Bu4N]Br (3)	4.5	45	59	1:0
7	[Bu_4_N]I (3)	4.5	15	52	1:0
8	Aliquat 336^[c]^ (3)	4.5	34	69	1:0
9	**A** (2)+[Bu_4_N]Cl (3)	4.5	83	78	1:0
10	**A** (2)+[Bu_4_N]I (3)	4.5	55	39	1:0
11	**A** (2)+[Bu_4_N]Cl (3)	24	93	74	1:0
12	**A** (2)+[Bu_4_N]Br (3)	24	96	55	1:0
13	**A** (2)+[Bu_4_N]I (3)	24	98	67	1:0
14	none	4.5	–^[d]^	–	–

[a] Reaction conditions: *trans*‐**2** (2.0—2.6 mmol) (see the Supporting Information IVc), 140 °C, 75 bar CO_2_ at 40 °C, biphenyl (10 mol %) as an internal standard, stirrer speed 300 rpm. [b] Determined from quantitative ^1^H NMR spectroscopy. [c] Aliquat 336=commercial mixture of C_8_ and C_10_ quaternary ammonium salts with predominately *N*‐methyl‐*N*,*N*,*N*‐trioctylammonium chloride. [d] 5 % mass loss.

Bromide is typically reported as the most efficient halide for cyclic carbonate synthesis due to its balanced nucleophilicity and leaving group character.^22^, ^27^, ^46^, ^52^, ^53^ However, for *trans*‐**3** synthesis we found chloride to be the most active and selective halide co‐catalyst, both on its own and in combination with **A** (Table 7). Activities decreased when moving to the less nucleophilic bromide and iodide. Given its corrosive and toxic nature, combined with reports of [Bu_4_N]F being ineffective in cyclic carbonate synthesis,^46^ fluoride was not tested. Sakakura reported that quaternary ammonium salts with longer alkyl chains were more active in cyclic carbonate synthesis;^43^ however, we found that substituting [Bu_4_N]Cl for Aliquat 336 (a commercial mixture of C8 and C10 quaternary ammonium salts) did not result in any significant change in activity (Table 7, entries 5 and 8).

### Integrated epoxidation and carbonation of 1,2‐limonene cyclic carbonate

**Figure 8:**
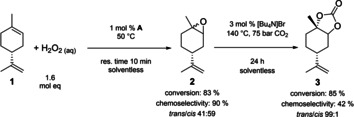
Two‐step synthesis of cyclic carbonates from limonene without intermittent epoxide isolation (res. time=residence time).

With an efficient catalyst system in hand, we explored the possibility of using **A** in a direct epoxidation–carboxylation sequence starting from terpenes. Limonene **1** was epoxidized with H_2_O_2_ using 1 mol % **A** to yield a 59:41 mixture of *cis*/*trans*‐**2** as previously reported.^38^ The resulting biphasic mixture was separated, and the organic phase was used directly in the CO_2_ insertion after addition of 3 mol % [Bu_4_N]Br co‐catalyst (Figure 8). Pleasingly, we found that, after 24 h, 85 % of **2** had reacted to **3** with 42 % chemoselectivity and 99 % stereoselectivity to *trans*‐**3**, which could be isolated in pure form by chromatography (crystallisation has been reported as an alternative purification method^21^, ^23^).

The overall yield of *trans*‐**3** across both steps starting from limonene of 39 % was mainly limited by the low epoxidation selectivity to *trans*‐**2**. Nevertheless, this is the first example of using the same catalyst for epoxidation and carbonation without intermittent epoxide isolation. To assess the effectiveness of our approach we compared it to reported methods for preparing **3**, including stereoselective stoichiometric epoxidations^54^ and some low‐pressure carbonation methods,^21^ in terms of process mass intensity (PMI) factors across both steps (Table 8). As can be seen from entries 1–5, although stereoselective oxidation provides almost exclusively *trans*‐**2** and consequently higher yield of *trans*‐**3**, the required intermediate workup of the epoxide leads to a 35 % higher PMI than our telescoped epoxidation/carbonation sequence catalysed by **A**, despite lower yields. Conventional routes to *trans*‐**3** involving classical mCPBA oxidation (entries 4 and 5) have the highest PMI due to the wasteful workup and non‐selective epoxide production.

**Table 8:** Calculated Process Mass Intensities (PMI) for various limonene epoxidation/carbonation systems.^[a]^

Entry	Reaction scheme	PMI^[b]^	Ref.
1^[c]^	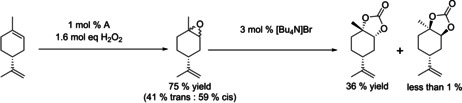	20	this work^[e]^
2	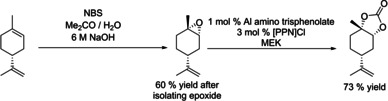	28	21, 54
3	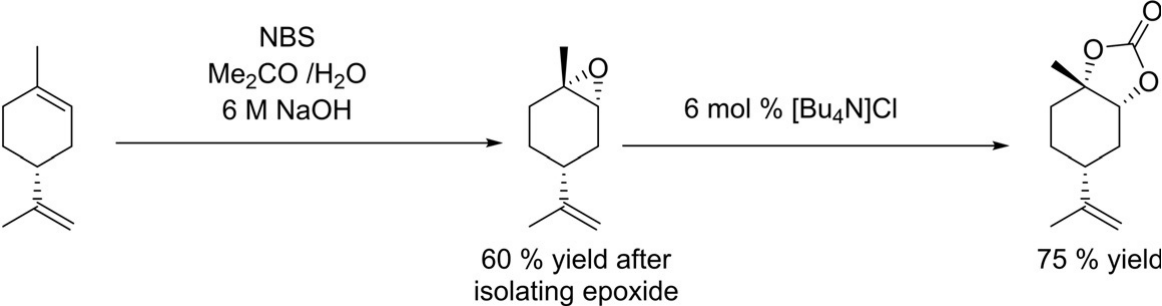	27	47, 54
4^[d]^	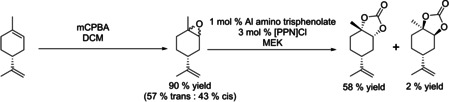	50	21
5^[d]^	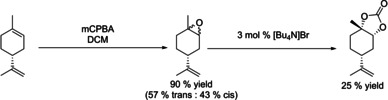	132	this work^[f]^

[a] All calculations performed for 5 mmol limonene, for details see the Supporting Information. [b] Carbonate isolation was not included in PMI calculations. [c] Calculations do not include waste generated during start‐up, before the flow reactor achieves steady state. [d] No data is available on commercial preparation of limonene oxide, mCPBA oxidation was chosen as an example: 5 mmol limonene, 5.5 mmol mCPBA, 5 mL DCM, assumed 90 % yield. [e] See Figure 8. [f] See Table 3, entry 2.

### Synthesis of 8,9‐limonene cyclic carbonate

Cyclic carbonate formation at the 1,2‐position of limonene is known to be facile due to the ease of selectively epoxidizing the more electron‐rich trisubstituted double bond. However, access to the corresponding 8,9‐epoxide and carbonate would provide an underexplored and potentially useful building block. The 8,9‐selectivity over 1,2‐functionalisation would most easily be achieved on the basis of steric differentiation. Indeed, Mizuno has reported [Bu_4_N]_4_[γHPV_2_W_10_O_40_] (**B**) as a bulky POM‐based catalyst for the selective epoxidation of limonene to 8,9‐limonene epoxide **4**.^55^ The epoxidation of limonene using **B** proceeded with high regioselectivity to **4** as a 6:4 mixture of two diastereoisomers. Pleasingly, we found CO_2_ insertion with **4** to be more facile than with the 1,2‐epoxides **2**. By using 3 mol % [Bu_4_N]Br at 140 °C, high conversions were achieved after only 2.5 hours (Table 9, entry 1). Lowering the temperature to 120 °C still gave good conversion with excellent selectivity of 99 % (entry 3). In contrast to **2**, **4** are less sterically hindered so that the nucleophilic attack is equally favourable on both isomers, and no significant difference in the isomer ratio of **4** was observed. While **A** was found to also catalyse the formation of **5**, the promoting effect of adding **A** to [Bu_4_N]Br was much lower than for **3** (entries 3–5). The attempted use of Mizuno's catalyst **B** for CO_2_ insertion yielded no cyclic carbonate but gave the 8,9‐limonene aldehyde **41** as the major product, irrespective of the amount of CO_2_ added (entries 7 and 8). Thus, unlike the 1,2‐selective epoxidation catalyst **A**, the 8,9‐selective epoxidation catalyst **B** does not catalyse carbonate formation and produces antagonistic effects in combination with halide co‐catalysts.

**Table 9:** Reaction of **4** with CO_2_.^[a]^

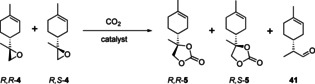								
Entry	Catalyst	*T* [°C]	*p* [bar]^[b]^	*t* [h]	*R*,*S*/*R*,*R* **4** after reaction	Conv. [%]^[c]^	Chemoselectivity **5** [%]	*R*,*S*/*R*,*R* **5^[^** ^d]^
1	[Bu_4_N]Br (3)	140	75	2.5	54:46	80	96	70:30
2	[Bu_4_N]Br (3)	120	75	4.5	58:42	76	91	65:35
3	[Bu_4_N]Br (3)	120	75	2.5	59:41	52	99	67:33
4	[Bu_4_N]Br (3)+**A** (2)	120	75	2.5	64:36	68	98	73:27
5	**A** (2)	120	75	2.5	64:36	46	86	83:17
6	[Bu_4_N]Br (3)+**B** (2)	120	75	2.5	61:39	57	76	63:37
7	**B** (2)	120	75	2.5	62:38	63^[e]^	0^[f]^	–
8	**B** (2)	120	–	2.5	56:44	75^[g]^	0^[h]^	–
9	no catalyst	120	75	2.5	63:37	–^[i]^	–	–

[a] Reaction conditions: **4** (2.3 mmol) (initial ratio of 63:37 *R*,*S*/*R*,*R*), biphenyl (10 mol %) as an internal standard, stirrer speed 300 rpm. [b] Pressure of CO_2_ at 40 °C. [c] Determined from quantitative ^1^H NMR spectroscopy. [d] From ^1^H NMR using doublet at 4.25 ppm, overlapping doublets were deconvoluted using MNova. [e] Up to 63 % depending on the batch of **B**. [f] Reaction yields **41** with 50 % selectivity. [g] Up to 75 % depending on the batch of **B**. [h] Reaction yields **41** with 47 % selectivity. [i] 6.5 % mass loss.

### Novel hydroxyurethanes from monofunctional cyclic carbonates

**Figure 9:**
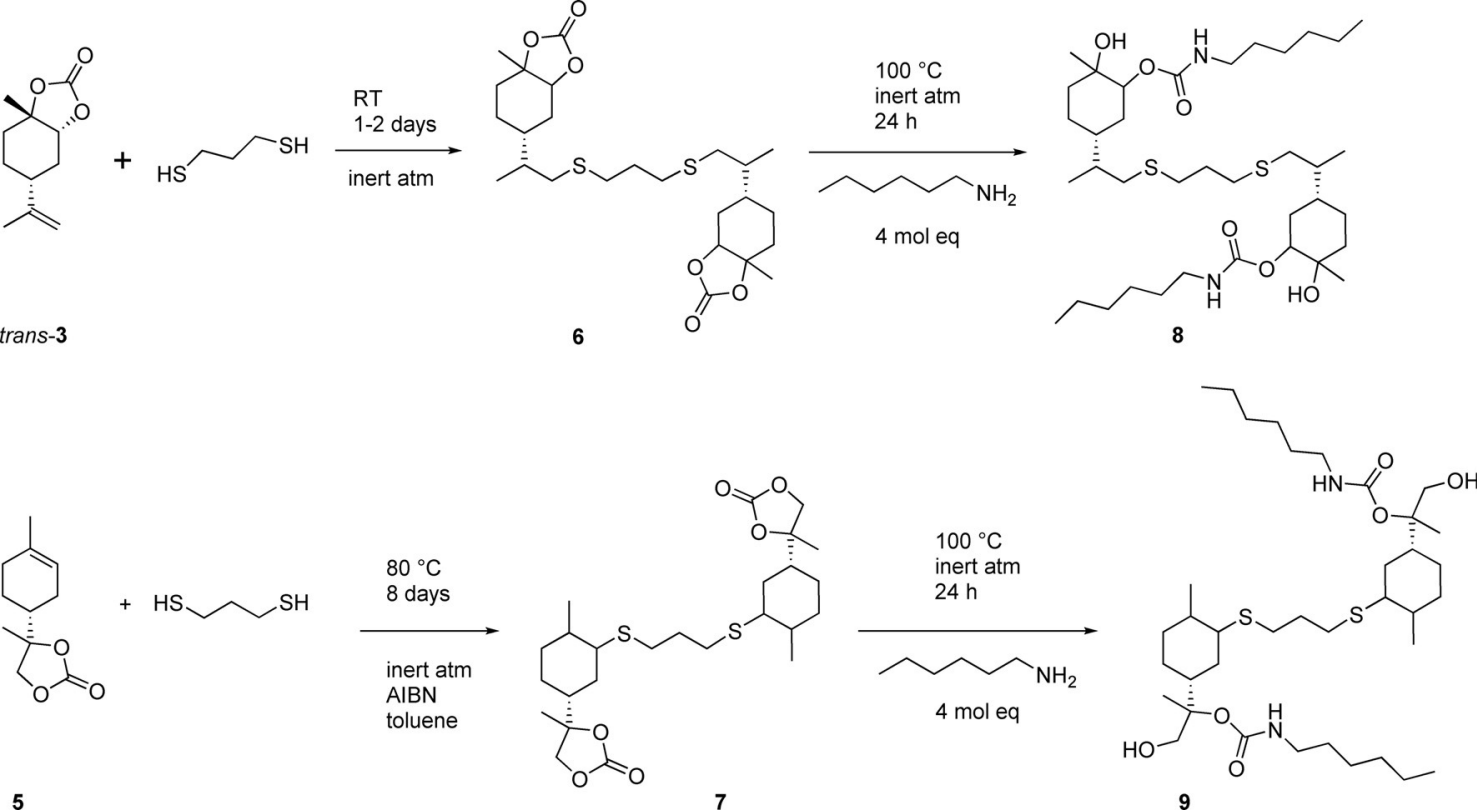
Synthesis of dithiol‐linked di‐monocarbonates from **3** and **5** via radical addition, and carbonate ring‐opening to hydroxyurethanes **8** and **9**.

In contrast to 1,2,8,9‐limonene carbonate, the use of monofunctional terpenoid cyclic carbonates **3** and **5** has remained unexplored so far, although they represent potentially useful building blocks for novel biorenewable NIPUs. Here we demonstrate their utility by linkage through a thiol–ene reaction on their remaining double bond to show that it is possible to create bifunctional carbonate dimers (**6** and **7**) that may be further ring opened to hydroxyurethanes using *n*‐hexylamine as a model substrate (Figure 9). Note that the sulfide linker is not required for actual NIPU synthesis and that the presented ring‐opening reaction serves as proof‐of‐concept and has not been optimised. Efficient procedures for selective carbonate ring‐opening have recently been published by Kleij and co‐workers.^56^

**Figure 10:**
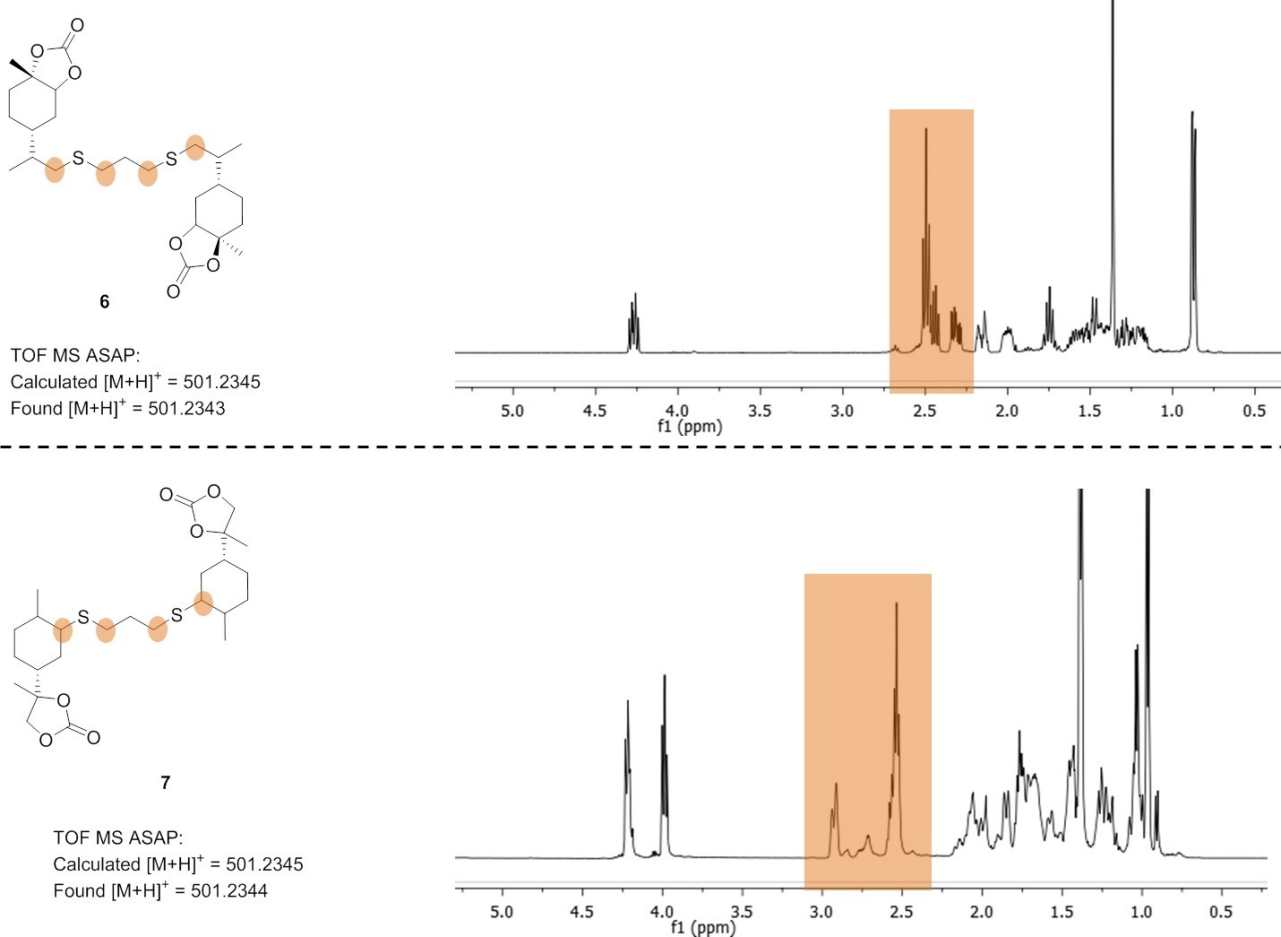
MS and ^1^H NMR spectroscopic analysis of **6** and **7** (further analytical details can be found in the Supporting Information).

Radical addition of di‐thiol on **3** to afford the linked di‐monocarbonate **6** could be carried out thermally without any exogeneous initiator.^14^ The endocyclic double bond in **5** however required addition of AIBN and longer reaction times to furnish the corresponding di‐monocarbonate **7**. The formation of **6** and **7** was confirmed by NMR spectroscopy and mass spectrometry (Figure 10).

**Figure 11:**
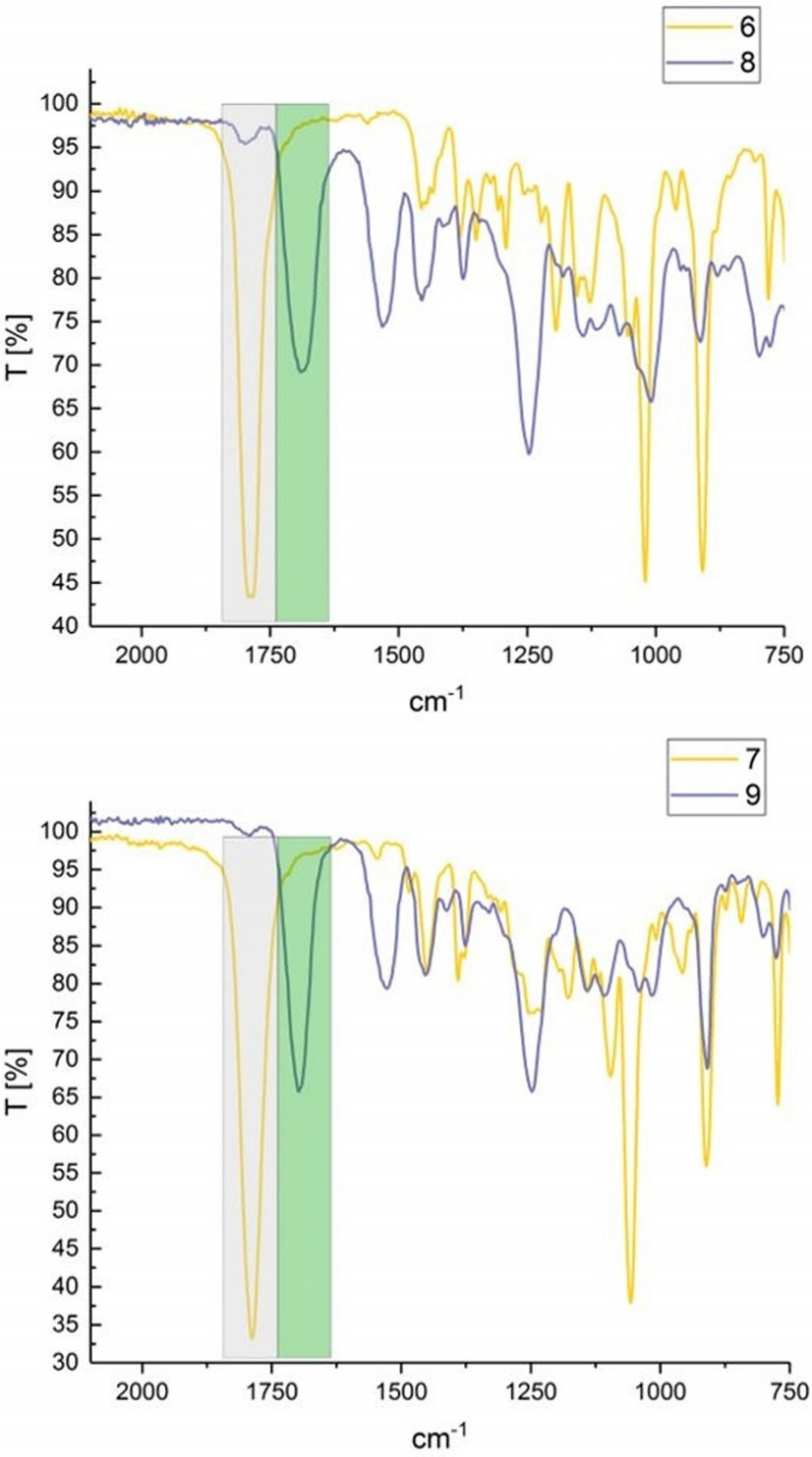
ATR‐IR spectra of **6** and **7** before (yellow) and after (blue) reaction with *n*‐hexylamine, showing the formation of novel hydroxyurethanes **8** and **9**, respectively. Carbonate C=O resonances are highlighted in grey, and amide C=O resonances in green.

Both bifunctional carbonate dimers **6** and **7** readily reacted with an excess of *n*‐hexylamine, resulting in ring‐opening of their cyclic carbonate functionalities after heating to 100 °C for 24 hours. NMR spectroscopic analysis of the reaction mixtures was challenging due to the number of regio‐ and stereoisomers present, but IR spectra showed complete consumption of cyclic carbonates in both cases through a characteristic shift of their carbonyl band at 1799 and 1792 cm^−1^ for **6** and **7**, respectively, to 1689 and 1698 cm^−1^ in the corresponding hydroxyurethanes **8** and **9** (Figure 11). Mass spectrometry confirmed the formation of **8** and **9** by revealing characteristic signals of [*M*+H]^+^ and [*M*+Na]^+^ adducts (additional data and details can be found in the Supporting Information).

The successful synthesis of hydroxyurethanes **8** and **9** demonstrates the potential of monofunctionalised terpene‐derived cyclic carbonates for renewable polymer synthesis: whereas the reaction of **6** and **7** with diamines would furnish linear polyurethanes, the reaction of **3** and **5** with polyfunctional amines would give access to pre‐polymers that may be further functionalised on the remaining double bond.^57^, ^58^, ^59^

## Conclusions

The growing interest in bio‐derived NIPUs and the commitment of the coating industry to improve their sustainability^60^ makes terpene‐derived cyclic carbonates desired compounds. 1,2,8,9‐Limonene biscarbonate has previously been used in NIPU synthesis to show promising results.^22^, ^23^ Here, we have synthesised limonene‐derived monocarbonates and their corresponding hydroxyurethanes by using benign and scalable catalytic methods. The synergistic interactions between the widely used Ishii‐Venturello epoxidation catalyst **A** and CO_2_ allowed for a telescoped two‐step synthesis of 1,2‐limonene cyclic carbonate directly from limonene. A different epoxidation catalyst (Mizuno's polyoxometalate **B**) selectively gave access to 8,9‐limonene oxide that could easily be transformed into its corresponding cyclic carbonate **5**, which was fully characterised for the first time. Both cyclic carbonates **3** and **5** have been shown to undergo thiol–ene reactions with 1,3‐propanedithiol to yield di‐monocarbonates **6** and **7** as versatile building blocks for polymer synthesis by nucleophilic ring‐opening with amines. Using combinations of various dithiols and polyamines with these limonene carbonates will give access to a range of novel NIPUs with tuneable properties and consisting of >50 wt % renewables. Application to other terpenes with less stereochemical restraints on the carbonation than limonene will give access to a wide range of bio‐based building blocks in even higher yields.

## Conflict of interest

The authors declare no conflict of interest.

## Supporting information

As a service to our authors and readers, this journal provides supporting information supplied by the authors. Such materials are peer reviewed and may be re‐organized for online delivery, but are not copy‐edited or typeset. Technical support issues arising from supporting information (other than missing files) should be addressed to the authors.

Supplementary
