# Supplementary material for: Selective Catalytic Synthesis of 1,2‐ and 8,9‐Cyclic Limonene Carbonates as Versatile Building Blocks for Novel Hydroxyurethanes
Source: Chemistry. 2020 May 8;26(33):7405–15. doi: 10.1002/chem.201905561 (PMC7317810; doi:10.1002/chem.201905561)
Supplement: Supplementary file 1 — Supplementary [file CHEM-26-7405-s001.pdf]

Chemistry–A European Journal

Supporting Information

Selective Catalytic Synthesis of 1,2- and 8,9-Cyclic Limonene Carbonates as Versatile Building Blocks for Novel Hydroxyurethanes

Katarzyna A. Maltby, Marc Hutchby, Pawel Plucinski, Matthew G. Davidson, and Ulrich Hintermair*[a]

Selective catalytic synthesis of 1,2- and 8,9-cyclic limonene carbonates as versatile building blocks for novel hydroxyurethanes

Katarzyna A. Maltby,[a] Marc Hutchby,[a] Pawel Plucinski,[a] Matthew G. Davidson,[a] Ulrich Hintermair*[a]

[a] *Centre for Sustainable and Circular Technologies, University of Bath, Claverton Down, BA2 7AY Bath, UK*
u.hintermair@bath.ac.uk

Supporting information

Table of Contents

I Materials and analysis 3

II Venturello catalyst (A) preparation 4

III The synthesis of 1,2-limonene oxide (2) using a microreactor 4

IV Kinetic resolution of 1,2-limonene oxide (2) 5

V The synthesis of 8,9-limonene oxide (4) using the Mizuno catalyst (B) 8

VI Cyclic carbonate synthesis using high pressure CO_2 10

VII Radical addition of 1,2-limonene cyclic carbonate (3) 10

VIII Radical addition of 8,9-limonene cyclic carbonate (5) 11

IX Hydroxyurethane synthesis 12

X Compounds characterisation 18

1. *Trans*-1,2-limonene oxide (*trans*-2) 18
2. *Cis*-1,2-limonene oxide (*cis*-2) 20
3. 8,9-limonene oxide (4) 21
4. *trans*-1,2-limonene cyclic carbonate (3) 23
5. 8,9-limonene cyclic carbonate (5) 25
6. Characterisation of compound 6 29
7. Characterisation of compound 7 32
8. 8,9-limonene aldehyde (41) 35

XI PMI calculations 37

XII References 43

I Materials and analysis

All reagents and NMR solvents were sourced from Sigma Aldrich, Alfa Aesar or Fisher Scientific. All solvents, except NMR solvents, were sourced from VWR and used as received. Liquid CO_2 was obtained from BOC (99.8 % purity).

GC-MS spectra were recorded on an Agilent system (GC: 7890B, MS: 5977A) using a high polarity nitroterephtalic acid-modified polyethylene glycol capillary column (Agilent Technologies DB-FFAP 30 m x 0.250 mm x 0.25) with a constant helium flow rate of 1.2 mL min^{-1} through column and a flame ionization detector (FID) held at 300 °C. Samples were prepared in toluene injected at 250 °C. The oven temperature increased by 20 °C min^{-1} from 40 °C to 250 °C where it was kept for 5.5 min.

NMR spectra were obtained on a Bruker Avance III 400 MHz, Agilent ProPulse 500 MHz, Bruker Avance III 500 MHz and Bruker Avance II + 500 MHz Ultrashield equipped with broadband observe probes. All NMR spectra were referenced against residual protic solvent peaks.

IR spectra were recorded on a Perkin Elmer 100 Fourier transform spectrometer fitted with an ATR accessory, accumulating four scans with 4 cm^{-1} resolution.

Mass spectrometry data was obtained from the EPSRC UK National Mass Spectrometry Facility at Swansea University and the University of Bath mass spectrometry facility using Bruker MicrOTOF electrospray time-of-flight mass spectrometer (ESI-TOF) and Bruker MaXis HD ESI-QTOF mass spectrometer for high mass accuracy.

High pressure experiments were carried out in glass inserts placed inside 10 mL stainless steel autoclaves equipped with a pressure gauge, a needle valve and 2-way valve. Stirring was provided using magnetic stir bar. The autoclaves were pressurised at 40 °C using a supercritical CO_2 installation schematically depicted on Figure 1. The pressurised autoclaves were then heated up to reaction temperature using aluminium heating blocks on a hotplate equipped with electronic thermometer.

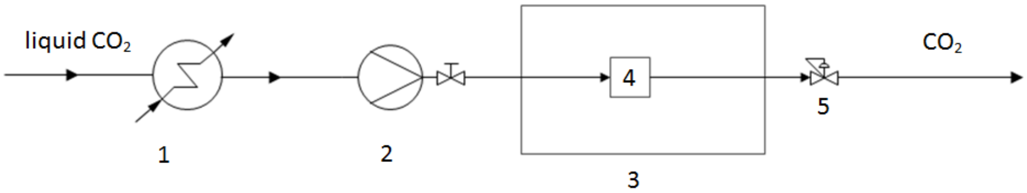

Figure 1 High pressure CO_2 instalation[1]. 1) cooler (Julabo FL300 Recirculating Cooler); 2) liquid CO_2 pump (JASCO PU-2088-CO2); 3) oven set at 40 °C; 4) 10 mL stainless steel autoclave; 5) back pressure regulator (JASCO BP-2080 plus)

II Venturello catalyst (A) preparation

$$4\ H_2WO_4 + 8\ H_2O_2 + H_3PO_4 \xrightarrow{H_2O} H_3PW_4O_{24} \quad \text{[Solution 1]}$$

$$3\ [(C_8H_{17})_3NCH_3]Cl + H_3PW_4O_{24} \xrightarrow{H_2O/CH_2Cl_2} [(C_8H_{17})_3NCH_3]_3(PW_4O_{24})$$

The Ishii-Venturello catalyst **A** for olefin epoxidation was prepared according to the original preparation method with minor changes.[2] 25 g tungstic acid was suspended in 80 mL 30 % wt hydrogen peroxide solution and stirred for 30 min at 70 °C leading to a colour change from yellow to light green. The light green solution was cooled to room temperature and 6 mL 40 % phosphoric acid were added, followed by 20 min stirring. Next, this solution was diluted with 220 mL distilled water and stirred for another 15-20 min (this solution is furthered referred as a Solution 1).

For the cation exchange we used the commercially available Aliquat 336 which is an ammonium salt mixture consisting mostly of methyltrioctylammonium chloride. 21 g Aliquat 336 was dissolved in 400 mL DCM and the resulting solution was added dropwise to Solution 1 under magnetic stirring followed by 30 min additional stirring. The resulting biphasic mixture was separated in a separating funnel. The organic phase was collected and washed with 2 x 150 mL of an aqueous solution saturated with NaCl. After washing, the organic phase was dried over $MgSO_4$, filtered and evaporated *in vacuo*. The yield of resulting yellow oil varied between 26 and 29 g (67 – 74 %).

III The synthesis of 1,2-limonene oxide (2) using a microreactor

Epoxidation was carried out according to previously published procedure[3] using a microreactor with preheating channels and an integrated static mixer (Little Things Factory, XXL-ST-04, 4.5 mL reaction volume). The reaction was carried out at 50 °C, with the temperature controlled by a recirculating heat exchanger (Fisher Scientific ISO UK 6200). The substrates were delivered to the reactor using syringe pumps. The first syringe was charged with 30 % wt hydrogen peroxide solution with the pH

adjusted to 7 using 1M NaOH. The second syringe was charged with pure limonene containing 1 mol % **A**. For the hydrogen peroxide solution, a plastic syringe was used. For the limonene and the catalyst solution, a stainless steel syringe was used. Both reactants were delivered to the reactor in equal volume ratios, with an overall flow rate of 27 mL/h (13.5 mL/h on each syringe), giving a 10 min residence time. Products were analysed by NMR and GC-MS. Conversion was 83 % and selectivity to **2** was 90 %. The reaction yielded a mixture of **2** isomers (59 % *cis*-**2** and 41 % *trans*-**2**).

Characteristic ^1H NMR signals: *trans*-**2** doublet at 2.98 ppm; *cis*-**2** multiplet at 3.03 ppm

IV Kinetic resolution of 1,2-limonene oxide (2)

a) *Cis*-1,2-limonene oxide (*cis*-2)

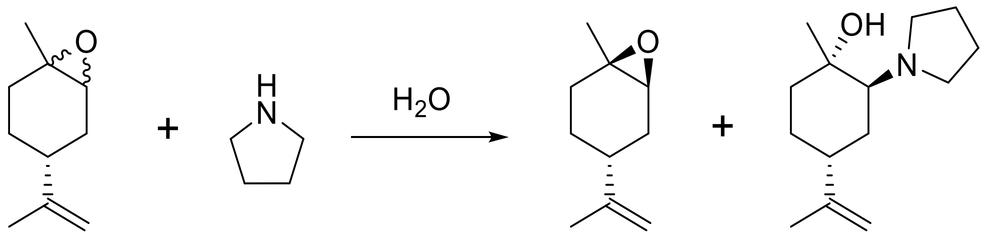

Figure 2 Kinetic resolution of **2** by selective opening of *trans*-**2**[4]

Cis-**2** was isolated from commercially available **2** following a literature procedure (Figure 2).[4] 16.4 mL **2**, 1.5 mL water and 8.35 mL pyrrolidine were placed in a 100 mL round bottom flask. The mixture was heated under reflux (100 °C) for 24 h. After cooling to room temperature, the mixture was dissolved in 50 mL diethyl ether (Et_2O) and washed twice with 50 mL water. The Et_2O was evaporated and *cis*-**2** was purified on a silica column chromatography using 20% Et_2O and 80 % petroleum ether 40-60 °C as a solvent. TLC plates were developed using a solution of phosphomolybdic acid in ethanol (EtOH). Yield after chromatography: 7 mL of colourless oil containing 96% *cis* isomer and 4 % *trans* isomer. 100 % *cis*-**2** can be obtained by repeating the procedure with a shorter reflux time of 1.5 h.

b) *Trans*-1,2-limonene oxide (*trans*-2)

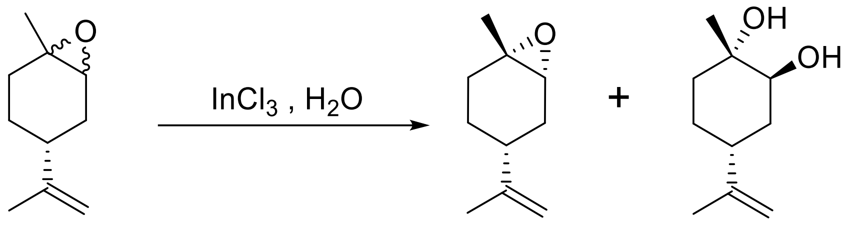

Figure 3 Kinetic resolution of **2** using indium chloride[5]

Following a previously reported method,[5] 0.664 g $InCl_3$ and 50 mL water were placed in a 100 mL round bottom flask. 4.5 g commercially available **2** (58 % *trans*-**2**) were added and the mixture stirred at room temperature for 24 h. After the reaction, the *trans*-**2** was extracted with DCM (3 x 50 mL). Finally, *trans*-**2** was purified using a column chromatography (20% Et_2O and 80 % petroleum ether 40-60 °C as a solvent, TLC plates were developed using a solution of phosphomolybdic acid in ethanol). Yield: 1.37 g of colourless oil consisting of 95% *trans*-**2** and 5 % *cis*-**2**. High purity (100 %) *trans*-**2** can be obtained by subjecting the product to the same procedure with a shorter reaction time of 8 h.

c) Large scale synthesis of *trans*-2

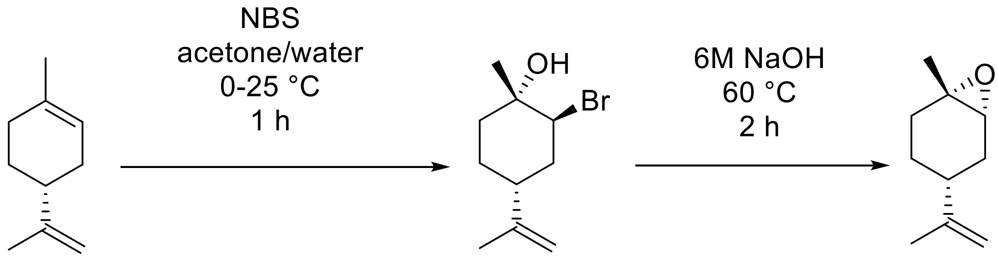

Figure 4 The synthesis of *trans*-**2** using NBS[6]

Following the literature procedure (Figure 4):[6] 60 mL limonene, 225 mL acetone, 50 mL water and a large magnetic stirrer bar were placed in a 500 mL round bottom flask and cooled in an ice bath. Then, 75 g N-bromosuccinimide (NBS) was added over 30 min followed by 1 h stirring. After the reaction, acetone was evaporated on a rotary evaporator. The bromohydrin was extracted with 150 mL Et_2O and separated from the aqueous phase, followed by washing with 60 mL water and evaporation of Et_2O. The bromohydrin was placed in a 250 mL round bottom flask with 100 mL 6 M aqueous solution of NaOH and stirred for 2 h at 60 °C. After the reaction, the mixture was left to cool

down and the NaOH solution was removed using a separating funnel. The remaining organic phase was dissolved in 150 mL Et_2O and washed with 45 mL saturated aqueous solution of $NaHCO_3$ and 45 mL water. After the evaporation of Et_2O, the resulting product is a yellow oil consisting 90 % **2** (95 % *trans*-**2**).

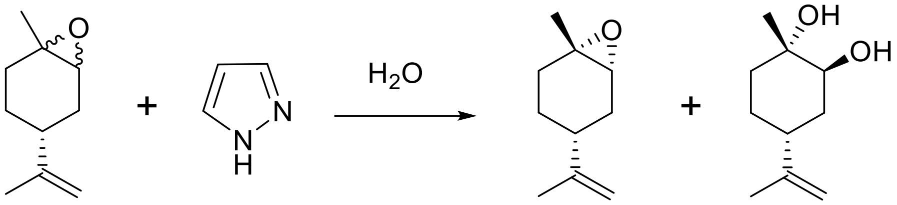

Figure 5 Selective hydrolysis of **2** yielding *trans*-**2**[4]

95 % *trans*-**2** was further purified following the literature procedure (Figure 5).[4] 20 g **2**, 1.50 g pyrazole and 70 mL water was placed in a 250 mL round bottom flask and was heated under reflux (100 °C) for 30 min. The reaction mixture was cooled down to 80 °C, placed in a separating funnel and washed twice with 150 mL water. The synthesised *trans*-**2** was purified by distillation yielding a 100 % *trans*-**2**; no *cis*-**2** was observed (90 % purity identified by GC-MS).

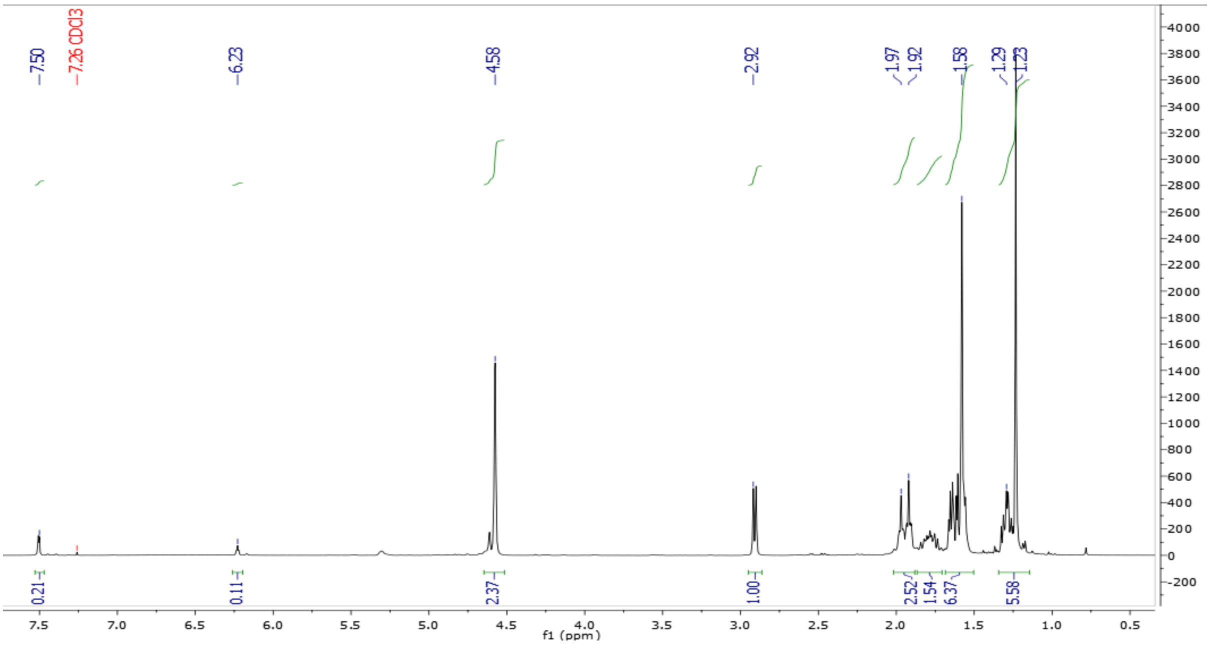

Figure 6 ^1H NMR spectrum of *trans*-**2** obtained from large scale synthesis. This batch of limonene oxide was used for experiments reported in Figure 6 and Table 4-6.

V The synthesis of 8,9-limonene oxide (4) using the Mizuno catalyst (B)

a) Mizuno catalyst preparation

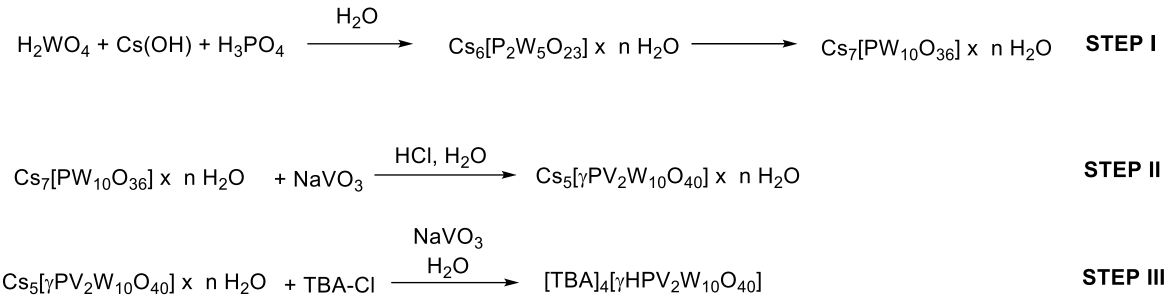

STEP I $Cs_7[PW_{10}O_{36}]$ x nH_2O preparation: Following the previously published method,[7] 6 g of tungstic acid was added to 40 mL water under magnetic stirrer resulting in a yellow slurry. 50 % wt. aqueous CsOH was added dropwise to the slurry under vigorous stirring until pH = 13 (about 11 mL CsOH solution). After CsOH addition, the mixture became cloudy, thus it was filtered over celite yielding a clear colourless filtrate. The pH of the filtrate was adjusted to 7 by adding 85 % H_3PO_4 dropwise (usually about 2.1 mL acid). The solution was stirred for 1 h, filtered again and cooled down in a refrigerator overnight. The resulting white solid ($Cs_6[P_2W_5O_{23}]$ x nH_2O) was recovered by filtration and refluxed in water for 24h. The mass ratio between the solid and water was 1:2 respectively. After reflux, the white solid ($Cs_7[PW_{10}O_{36}]$ x nH_2O) was separated by filtration.

Note: The quality of the CsOH solution used has a crucial impact on the synthesis. The reported procedure was carried out using Alfa Aesar™ Caesium hydroxide (50% w/w aq. soln., 99% metals basis) handled and stored air-free.

STEP II $Cs_5[\gamma PV_2W_{10}O_{40}]$ x nH_2O preparation: Following the previously published method,[7-9] 0.1 g sodium metavanadate ($NaVO_3$) was dissolved in 4 mL water by heating to 70 °C. The solution was cooled down to room temperature and 3 M HCl was added dropwise to adjust the pH to 0.8. The solution became yellow, which indicated the presence of $[VO_2]^+$. 1.25 g $Cs_7[PW_{10}O_{36}]$ was slowly added to the yellow solution. The solution was stirred for an additional 30 min and filtered yielding a yellow solid ($Cs_5[\gamma PV_2W_{10}O_{40}]$ x nH_2O). The resulting yellow solid (γ isomer) is stable at pH 2 and in the presence of $[VO_2]^+$. In the absence of $[VO_2]^+$ isomerisation to β-isomer occurs which has an orange colour.[7]

STEP III [TBA]$_4$[γHPV$_2$W$_{10}$O$_{40}$] preparation: Following the previously published method,[10] 0.145 g of NaVO$_3$ was dissolved in 120 mL water at 70 °C, cooled down to room temperature and acidified to pH 2 using 3 M HCl (about 0.8 mL 3 M HCl was needed). 3.8 g Cs$_5$[γ-PV$_2$W$_{10}$O$_{40}$] was added. The resulting solution was filtered in order to remove insoluble solids, and 1.55 g TBA-Cl was added in one portion. The reaction was stirred for 10 min and filtered, yielding a yellow solid which will be referred to as the Mizuno catalyst (**B**). IR analysis results were in accordance with previously published data.[7, 10]

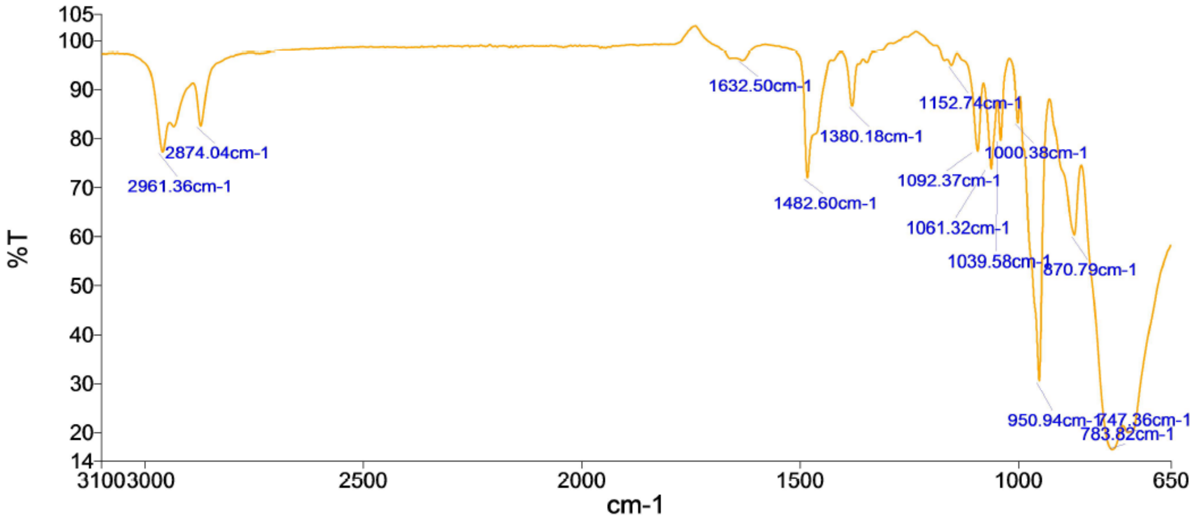

Figure 7 IR of the final Mizuno catalyst [TBA]$_4$[γHPV$_2$W$_{10}$O$_{40}$] (**B**)

b) limonene epoxidation using B

The epoxidation of limonene to 8,9-limonene oxide (**4**) was conducted according to the previously reported method.[10] The 0.1 mmol **B** (358 mg), magnetic stirrer bar and a 60 mL mixture of acetonitrile (MeCN) and tert-butanol (t-BuOH) (1:1 volume) was placed in a 100 mL round bottom flask. The catalyst was activated by adding 14 mg of 70% HClO$_4$ (about 1-2 drops). Finally, 20 mmol limonene (3.2 mL) was added to the round bottom flask. The mixture was warmed up to 32 °C and a 30 % hydrogen peroxide solution was added to the reaction mixture in 5 portions every 10 min (5 x 0.41 mL). After complete hydrogen peroxide addition, the reaction mixture was stirred for 30 min. After the reaction, organic solvents were removed *in vacuo* until precipitate of catalyst was formed. 60 mL Et$_2$O was added to the resulting mixture and **4** was filtered from precipitate followed by evaporation of Et$_2$O. The resulting oil was purified by a silica column chromatography using a mixture of Et$_2$O and petroleum spirit as a solvent (2:98 Et$_2$O : petroleum spirit) . TLC plates were

developed using solution of phosphomolybdic acid in EtOH. The obtained product was a mixture of 8,9-limonene oxide stereoisomers 36% 4*R*,8*R*-**4** and 64% 4*R*,8*S*-**4**.[11]

VI Cyclic carbonate synthesis using high pressure CO_2

High pressure cyclic carbonate synthesis was performed in a 10 mL stainless steel autoclave with a glass liner. Epoxide, biphenyl and catalyst were placed in the glass liner, which was placed in the pre-heated to 40 °C autoclave. The autoclave was sealed and pressurised with CO_2 to the desired pressure at 40 °C and then the autoclave was placed in an aluminium heating block, preheated to the reaction temperature. After the reaction, the autoclave was cooled to room temperature and the pressure was slowly released over 20 minutes. Conversion and selectivity were calculated by ^{1}H NMR using biphenyl as an internal standard.

VII Radical addition of 1,2-limonene cyclic carbonate (3)

a) The synthesis of 6

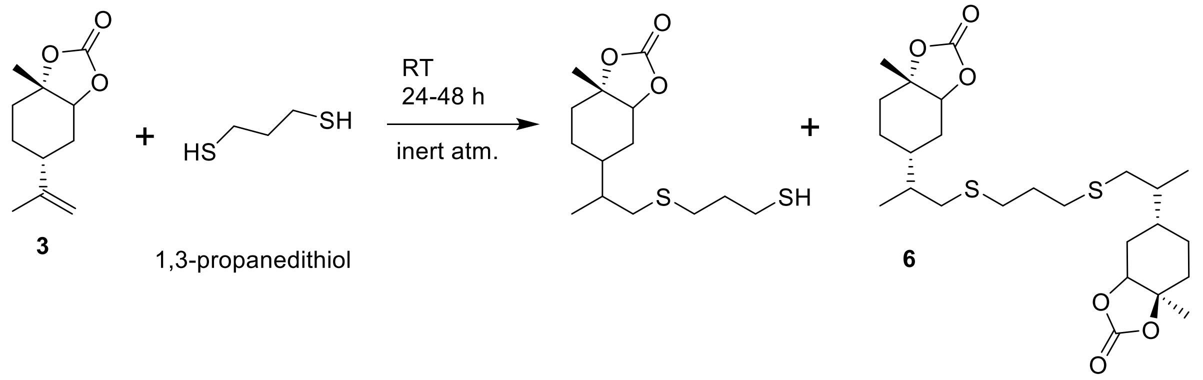

Figure 8 Synthesis of compound **6**

The synthesis of **6** was performed using method developed by Meier *et al.* which does not require a radical initiator.[12] 2.076 g (10.59 mmol) of **3** was placed in a round bottom flask and put under inert atmosphere of Argon. Then, 0.53 mL of 1,3-propanedithiol was added to the flask and flask was flushed with Argon. The reaction was stirred at room temperature for 24 h. The progress of reaction can be monitored by ^{1}H NMR using the signal from double bond at 4.75 ppm. After 24 h, 80 % of **3** reacted. To fully react the remaining **3** another 0.53 mL of 1,3-propanedithiol was added and stirred for another 24 h.

The crude reaction mixture was purified by silica column chromatography using mixture of ethyl acetate and petroleum spirit as a solvent (40:60 ethyl acetate : petroleum spirit). Isolated yield of **6**: 1.568 g (3.13 mmol), 59 %.

VIII Radical addition of 8,9-limonene cyclic carbonate (5)

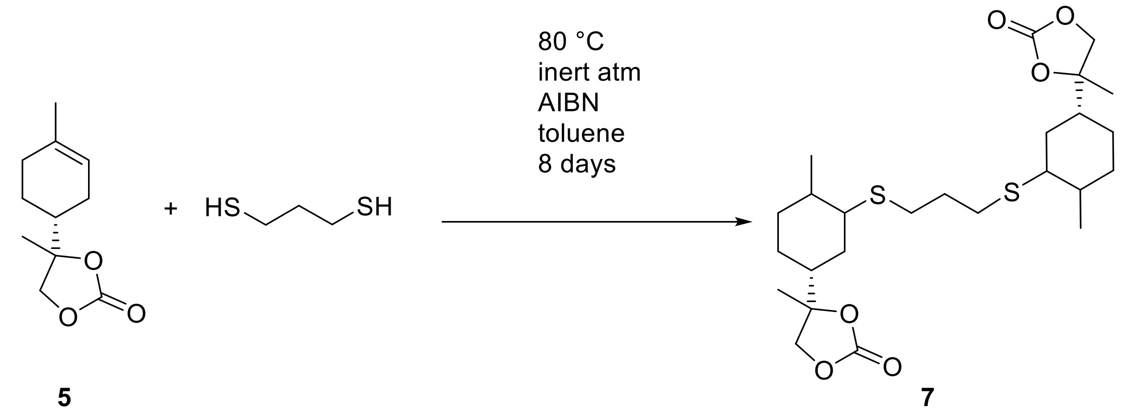

Figure 9 Synthesis of compound **7**

The double bond in **5** is less reactive than the double bond in **3**, thus the method without radical initiator did not yield the desired product.

2.741 g (13.4 mmol) **5** was placed in a round bottom flask and put under inert atmosphere of Argon. 0.229 g AIBN was dissolved in 7 mL dry toluene and added to **5**. The flask was flushed with Argon a few times and 0.7 mL 1,3-propanedithiol was added to the mixture in the flask. The reaction was stirred for 8 days at 80 °C. After this time 72 % of **5** reacted.

The reaction mixture was purified using silica column chromatography using ethyl acetate and petroleum spirit (40:60 respectively) as a solvent. Isolated yield of **7**: 1.169 g (2.34 mmol), 35 %

IX Hydroxyurethane synthesis

a) Synthesis of compound 8

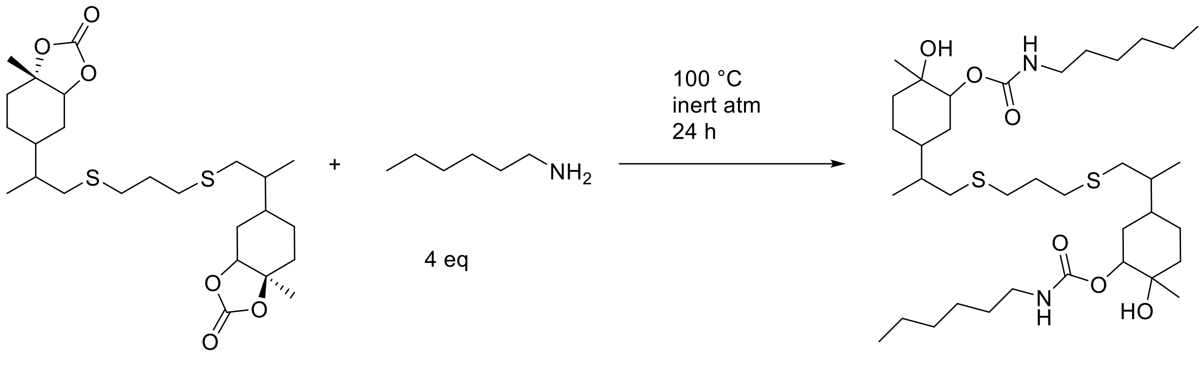

6

8

Figure 10 Urethane synthesis from **6**

Note: This reaction will yield a mixture of isomers, for clarity only one is presented.

0.432 g of **6** and magnetic stirrer bar was placed in round bottom flask attached to reflux condenser. **6** was put in the inert atmosphere of argon. 0.5 mL of hexyl amine (4 molar equivalents) was added to the round bottom flask and the temperature was increased to 100 °C. The reaction was left for 24 h. After the reaction, the excess of amine was evaporated *in vacuo*. The resulting yellow-brown oil was analysed by NMR, mass spectrometry and FT-IR.

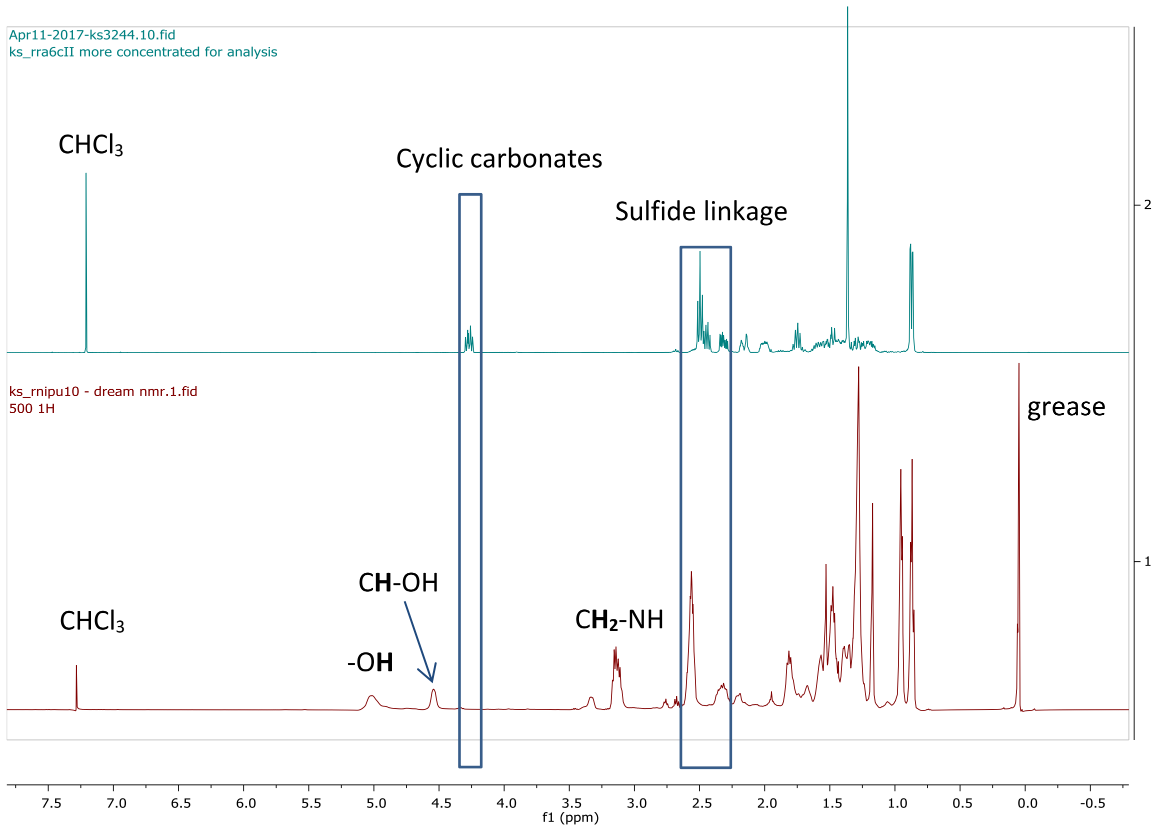

Figure 11 ^{1}H NMR of **6** (top) and **8** (bottom). **8** was not further purified, the presented spectrum is a spectrum of crude reaction mixture after evaporating an excess of amine.

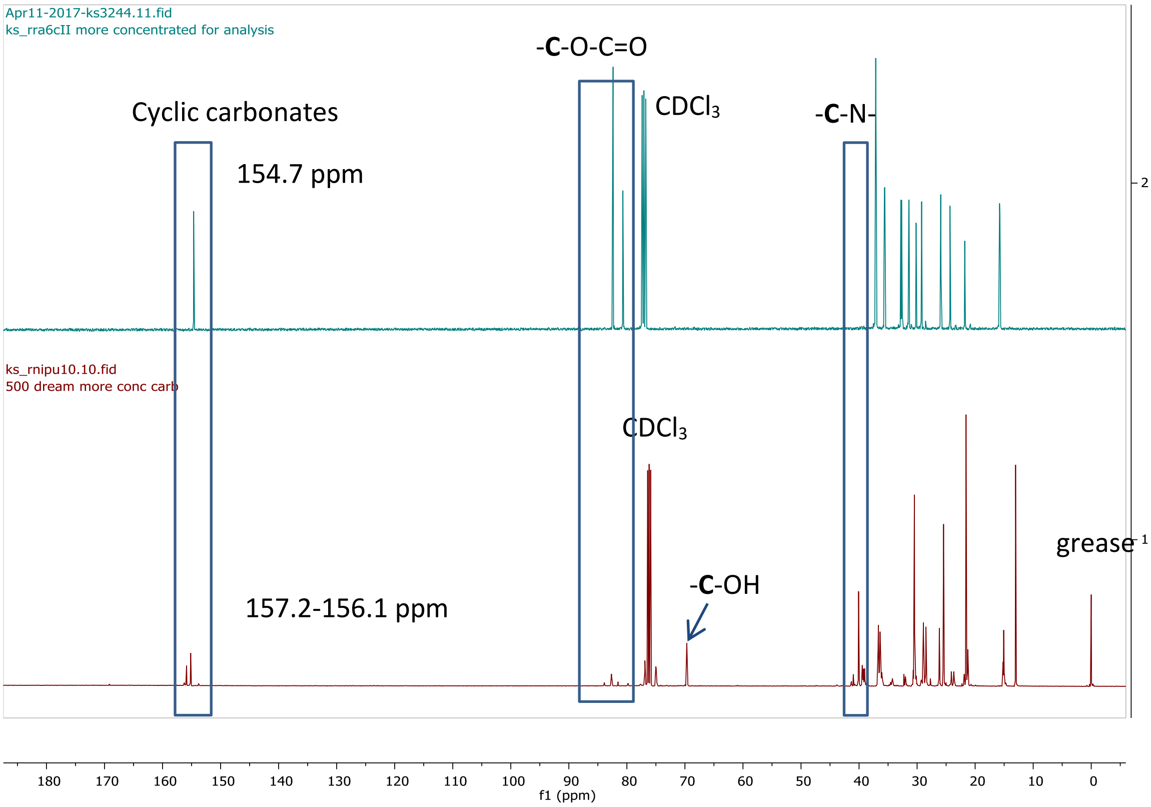

Figure 12 ^{13}C {^{1}H} NMR of **6** (top) and **8** (bottom). **8** was not further purified, the presented spectrum is a spectrum of crude reaction mixture after evaporating an excess of amine.

MS ESI-TOF: Calculated $[M+H]^+$ = 703.4748, $[M+Na]^+$ = 725.4573. Found $[M+H]^+$ = 703.4752, $[M+Na]^+$ = 725.4690.

FT-IR: 1689 cm^{-1} (C=O)

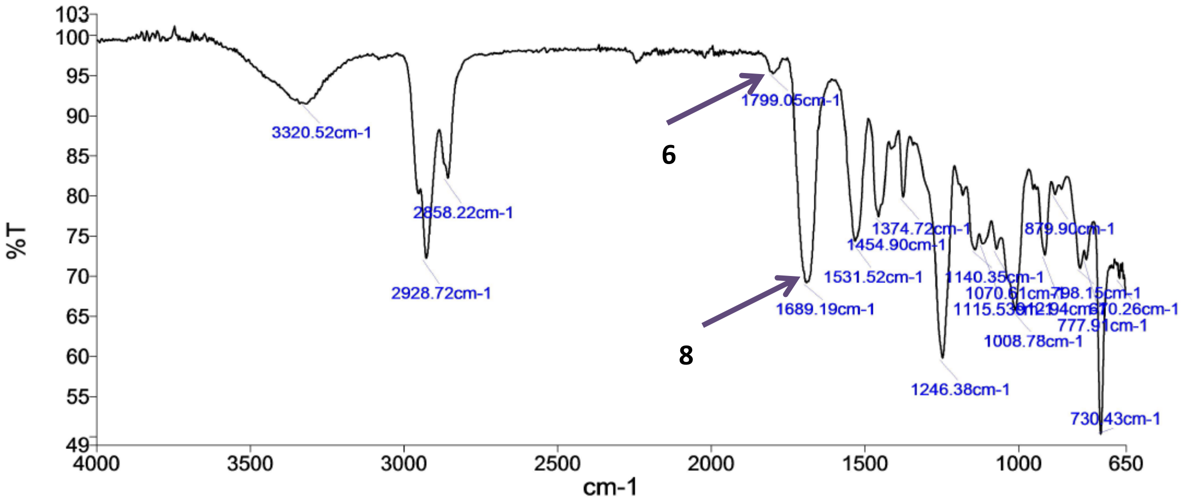

Figure 13 IR of crude reaction mixture from the synthesis of **8** after evaporation of the excess of amine

b) Synthesis of 9

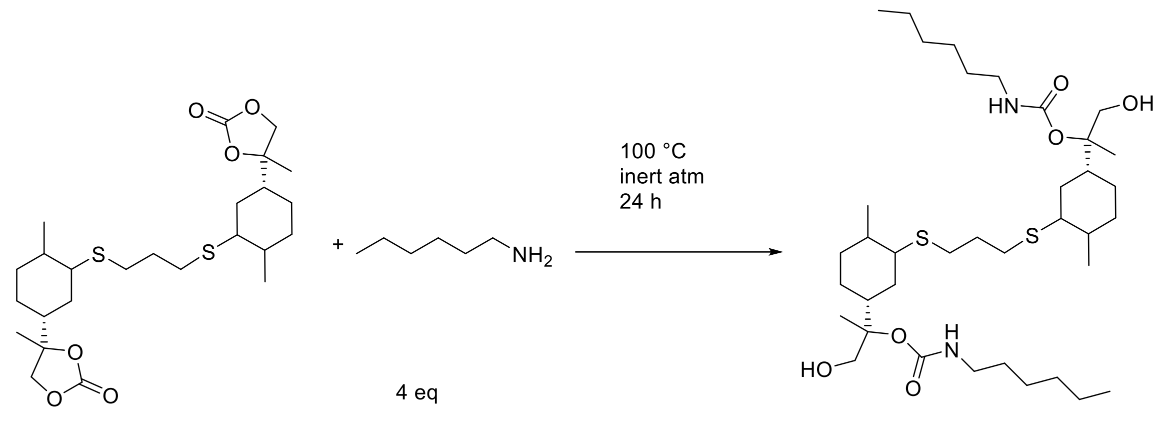

7

9

Figure 14 Urethane synthesis from **7**

Note: This reaction will yield a mixture of isomers, for clarity only one is presented.

0.739 g of **7** and magnetic stirrer bar was placed in round bottom flask attached to reflux condenser. **7** was put in the inert atmosphere of argon. 0.8 mL of hexyl amine (4 molar equivalents) was added to the round bottom flask and the temperature was increased to 100 °C. The reaction was left for 24 h. After the reaction, the excess of amine was evaporated *in vacuo*. The resulting yellow-brown oil was analysed by NMR, mass spectrometry and FT-IR.

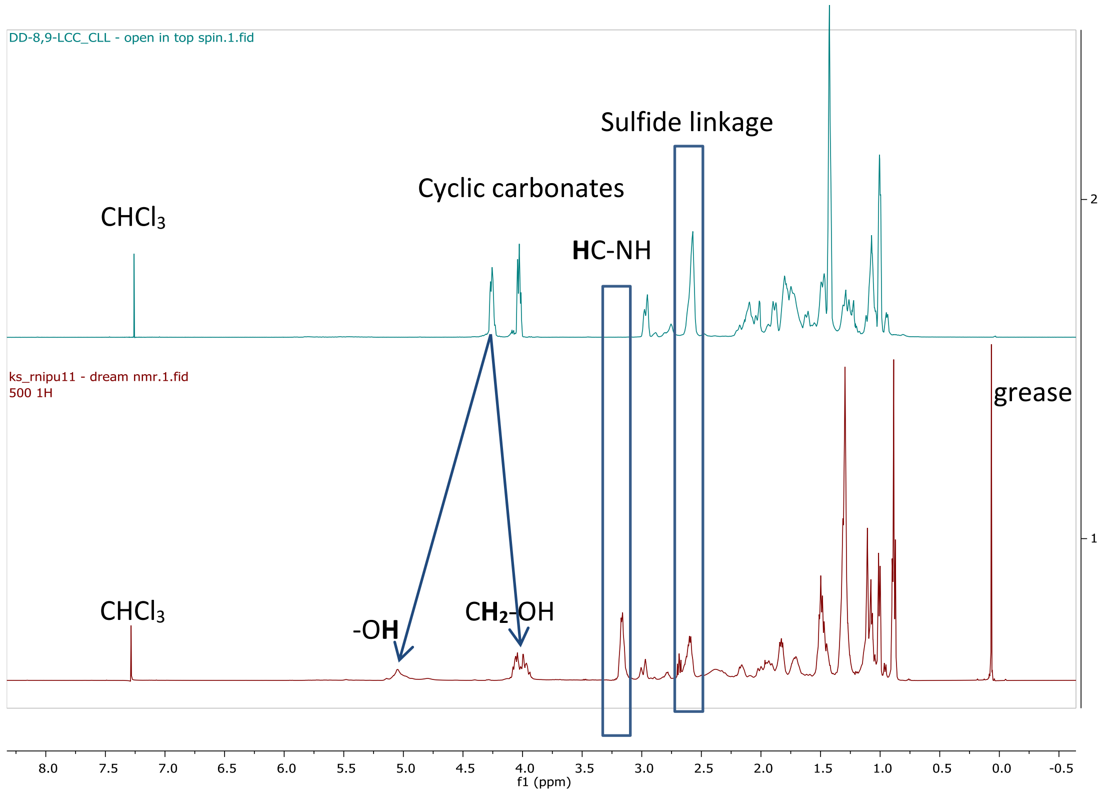

Figure 15 ^{1}H NMR of **7** (top) and **9** (bottom). **9** was not further purified, the presented spectrum is a spectrum of crude reaction mixture after evaporating an excess of amine.

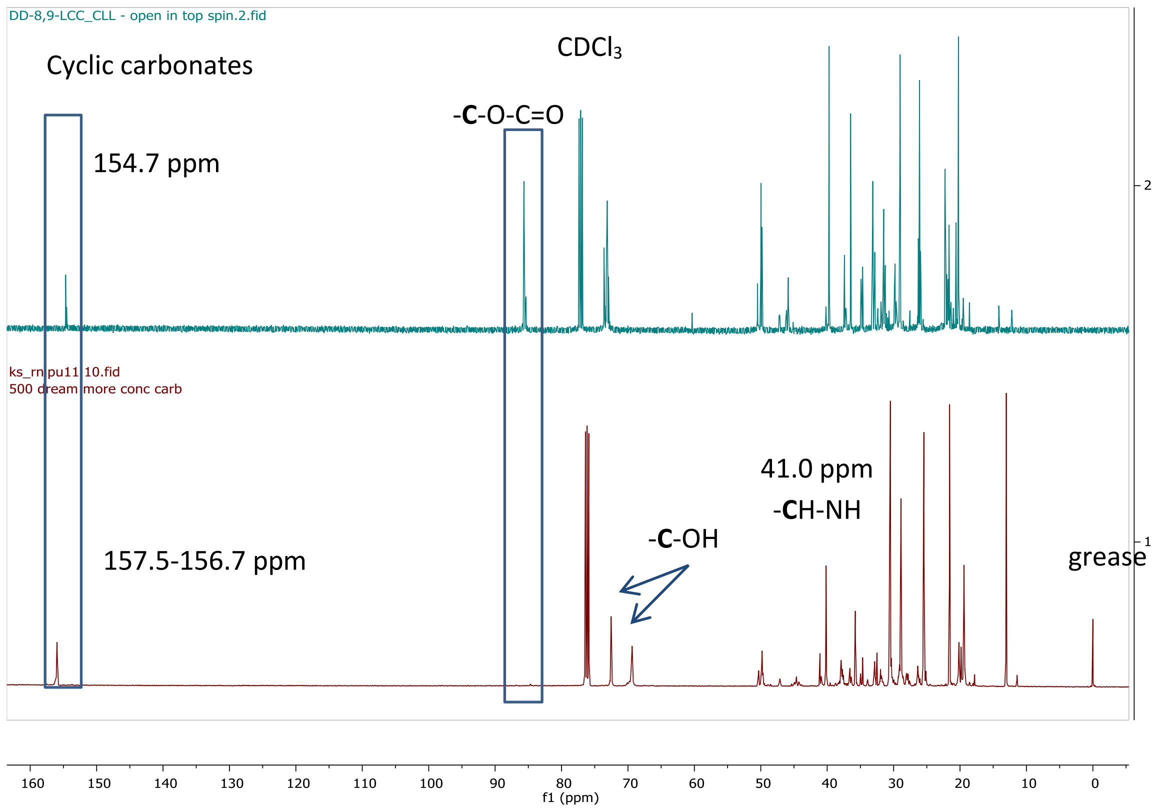

Figure 16 ^{13}C {^{1}H} NMR of **7** (top) and **9** (bottom). **9** was not further purified, the presented spectrum is a spectrum of crude reaction mixture after evaporating an excess of amine.

MS ESI-TOF: Calculated $[M+H]^+$ = 703.4748, $[M+Na]^+$ = 725.4573. Found $[M+H]^+$ = 703.4774, $[M+Na]^+$ = 725.4624.

FT-IR: 1698 cm^{-1} (C=O)

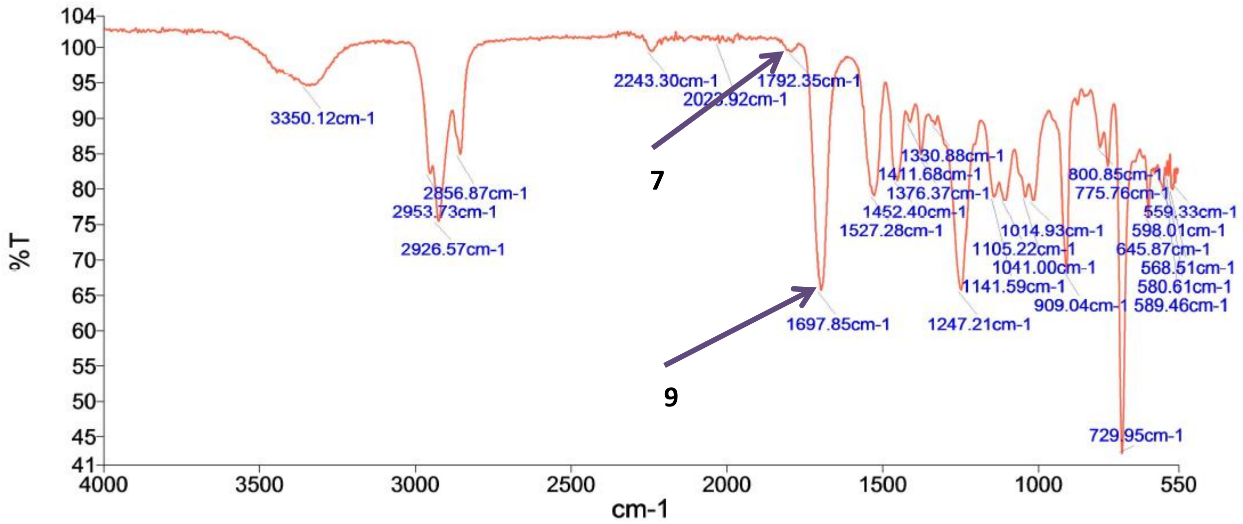

Figure 17 IR of crude reaction mixture from the synthesis of **9** after evaporation of the excess of amine.

X Compounds characterisation

1. *Trans*-1,2-limonene oxide (*trans*-2)

trans-2

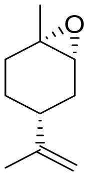

Trans-**2** is a previously known compound [4, 13]

^{1}H NMR (400 MHz, $CDCl_3$) δ = 4.76 – 4.59 (m, 2H), 2.99 (d, $^{3}J_{H\text{-}H}$ =5.4 Hz, 1H), 2.20 – 1.97 (m, 2H), 1.93 – 1.79 (m, 1H), 1.76 – 1.61 (m, 3H+2H), 1.48 – 1.12 (m, 3H+2H).

^{13}C {^{1}H} NMR (101 MHz, $CDCl_3$) δ = 149.2, 109.2, 59.3, 57.5, 40.8, 30.9, 30.0, 24.4, 23.2, 20.3.

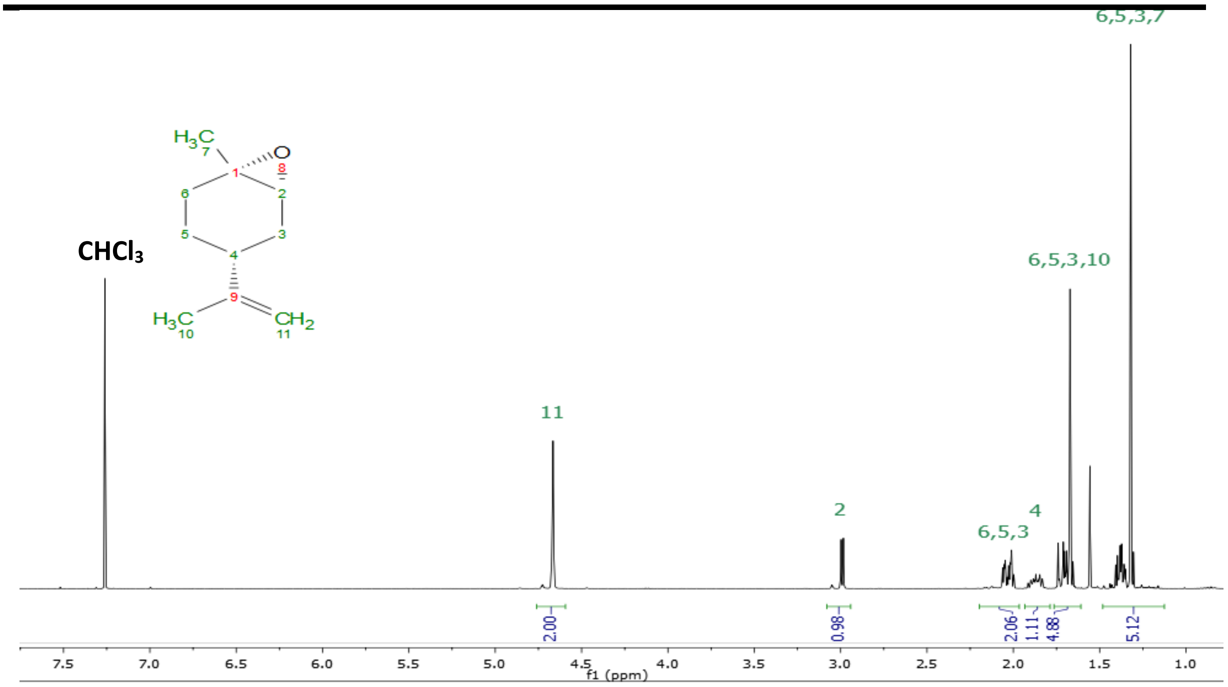

Figure 18 ^{1}H NMR of 95 % *trans*-**2**

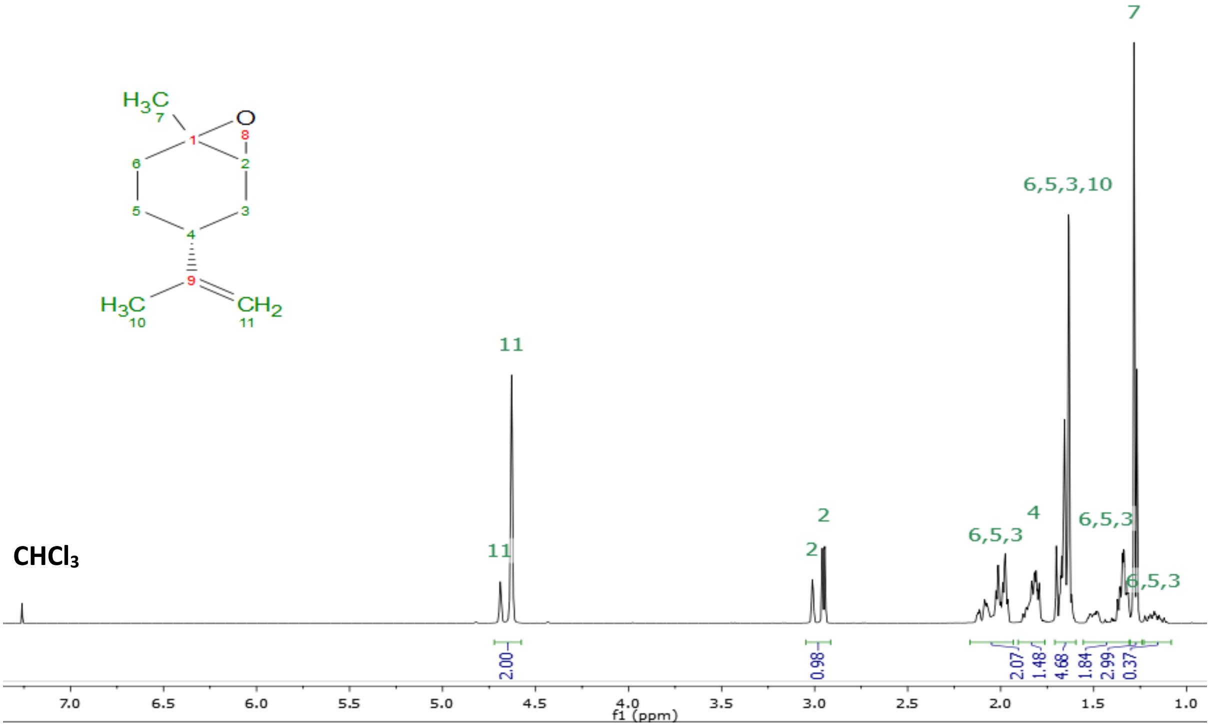

Figure 19 ^{1}H NMR of **5**, 70 % *trans*-**2** 30 % *cis*-**2**

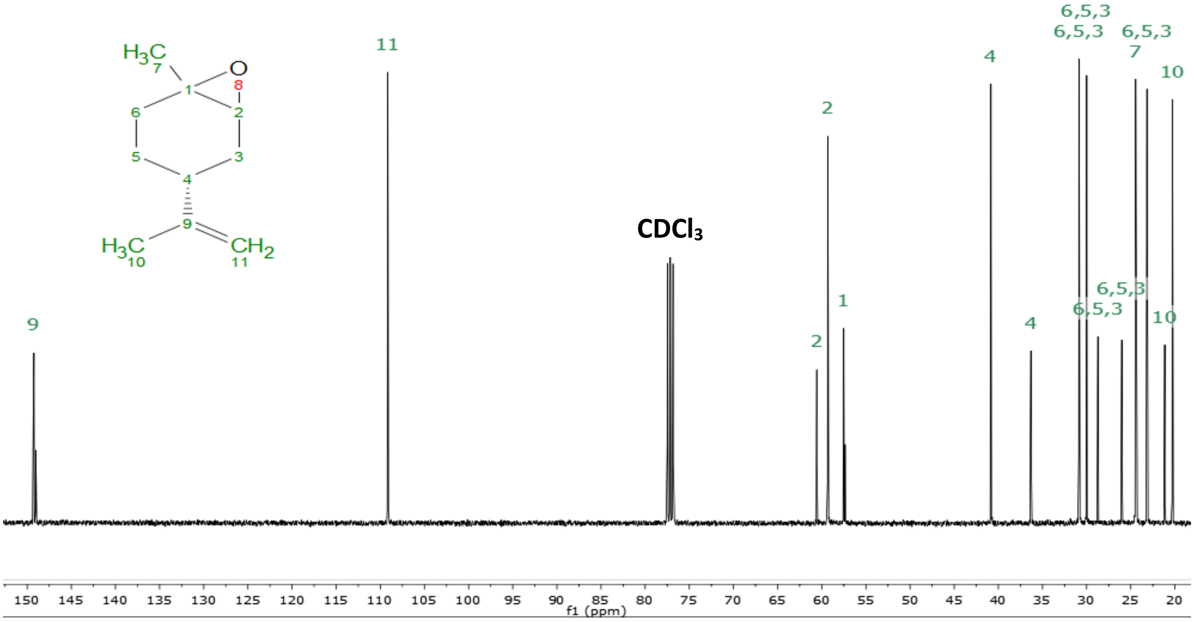

Figure 20 ^{13}C {^{1}H} NMR of **2** 70 % *trans*-**2** 30 % *cis*-**2**

2. *Cis*-1,2-limonene oxide (*cis*-2)

cis-**2**

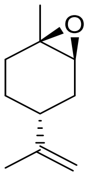

Cis-**2** is previously known compound[4, 13]

^{1}H NMR (400 MHz, $CDCl_3$) δ = 4.73 – 4.58 (m, 2H), 3.04 – 2.91 (m, 1H), 2.15 – 1.93 (m, 2H), 1.87 – 1.72 (m, 2H), 1.72 – 1.56 (m, 4H), 1.54 – 1.31 (m, 1H), 1.30 – 1.23 (m, 3H), 1.24 – 1.06 (m, 1H).

^{13}C {1H} NMR (101 MHz, CDCl3) δ = 149.0, 109.1, 60.5, 57.3, 36.3, 30.8, 28.7, 26.0, 24.3, 21.1.

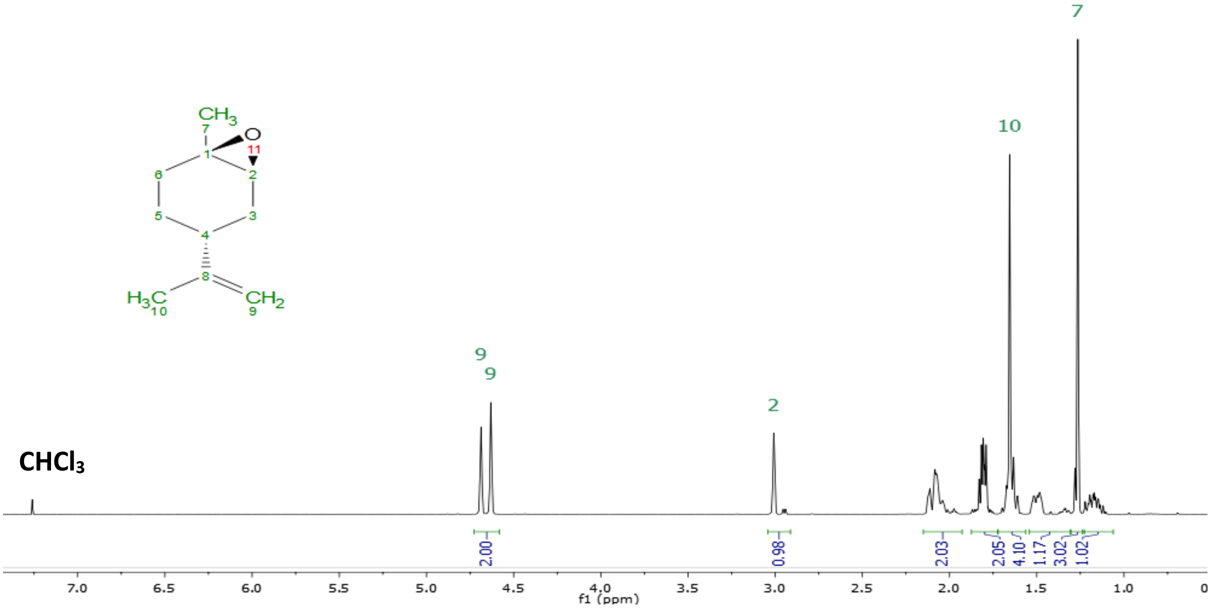

Figure 21 ^{1}H NMR of 92 % *cis*-**2**

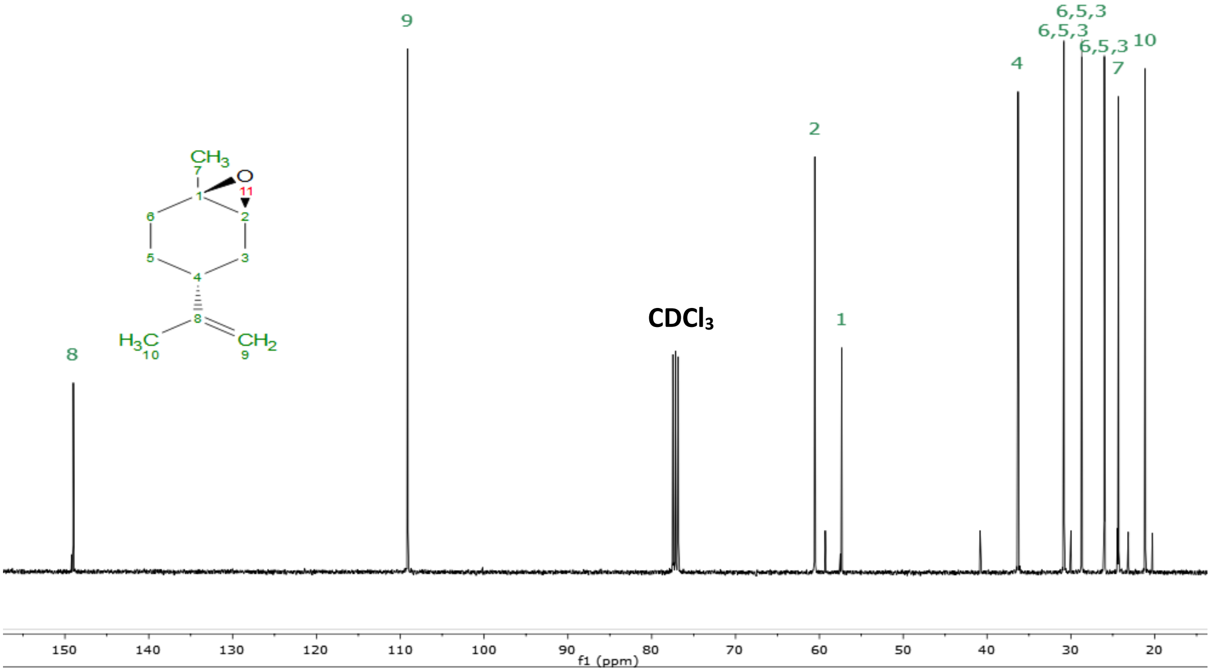

Figure 22 ^{13}C {^{1}H} NMR of 92 % *cis*-**2**

3. 8,9-limonene oxide (4)

8,9-limonene oxide (4)

4*R*,8*R*-4 | **4*R*,8*R*-4**

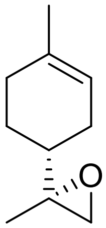

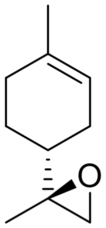

4 is a previously known compound[10-11]

mixture of isomers:

^{1}H NMR (400 MHz, $CDCl_3$) δ = 5.32 (m, 1H), 2.62 – 2.57 (m, 1H), 2.53 – 2.47 (2xd, 1H), 1.93 (m, 5H), 1.60 (m, 3H), 1.22 (m, 5H).

^{13}C {1H} NMR (101 MHz, $CDCl_3$) δ = 134.1, 133.9, 120.1, 119.9, 59.4, 59.2, 53.4, 52.7, 40.2, 39.7, 30.4, 30.1, 27.5, 27.5, 25.0, 24.9, 23.5, 23.4, 18.8, 18.1.

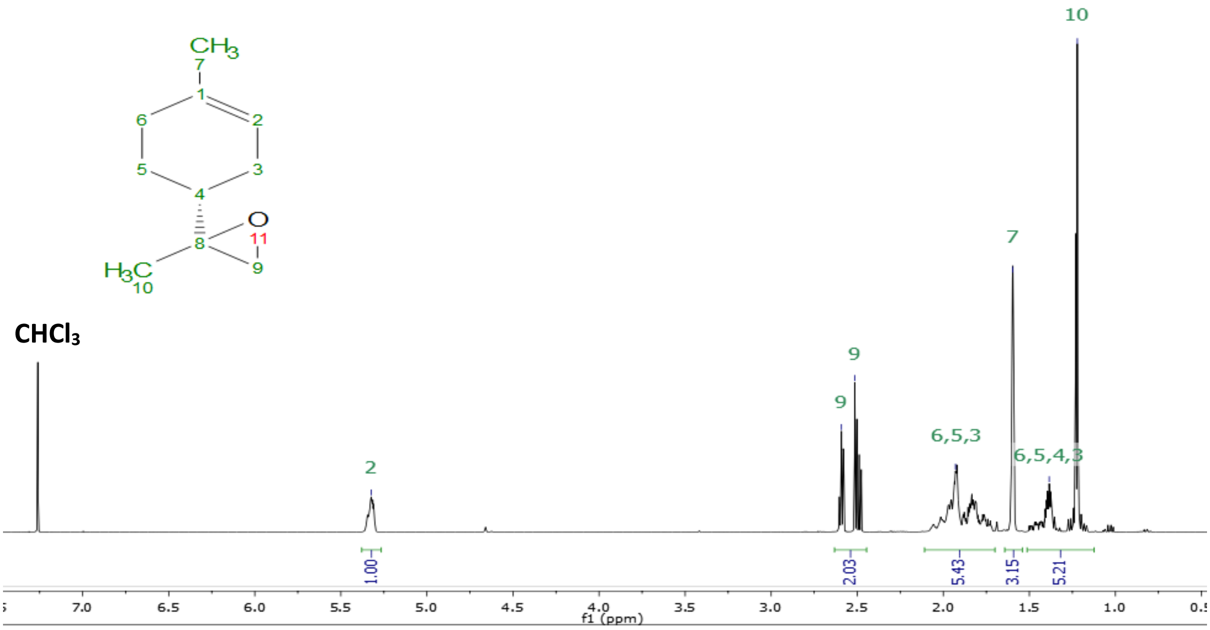

Figure 23 ^{1}H NMR of **4**

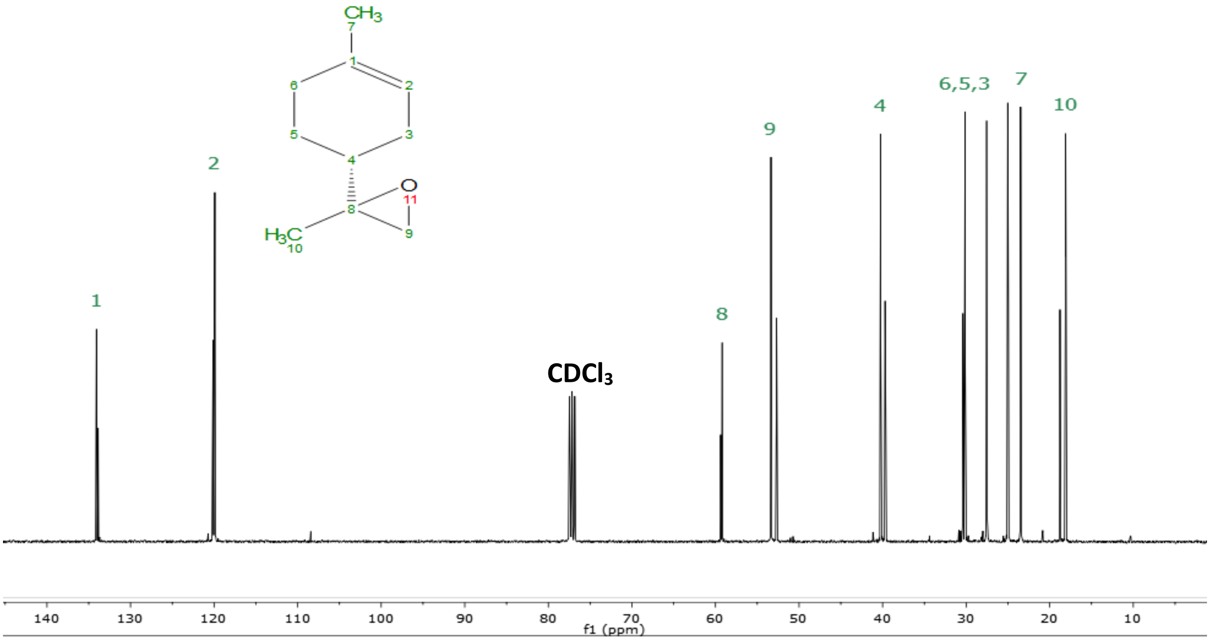

Figure 24 ^{13}C {^{1}H} NMR of **4**

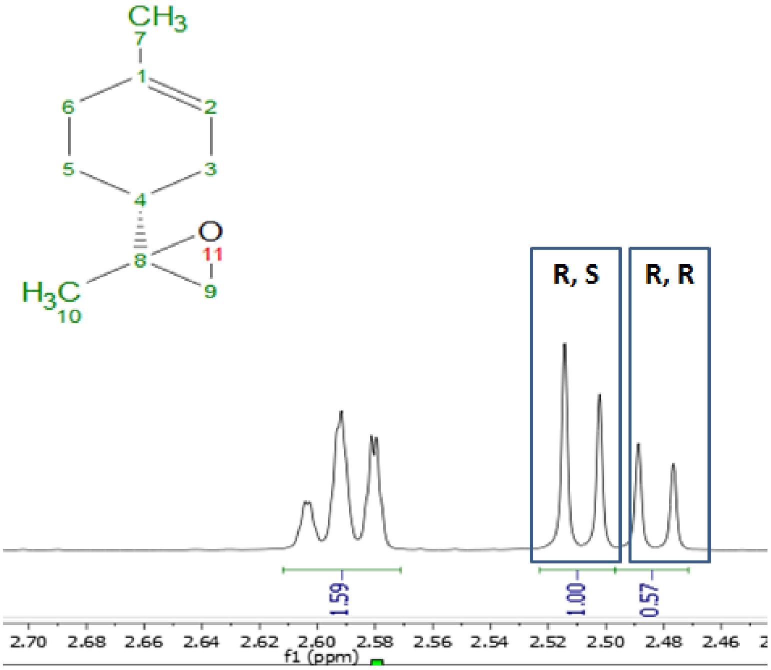

Figure 25 ^{1}H NMR spectra of protons in position 9 with characteristic stereoisomers peaks[11]

4. *trans*-1,2-limonene cyclic carbonate (3)

trans-1,2-limonene cyclic carbonate (_trans_-3)

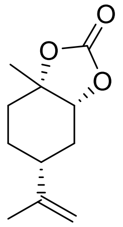

Trans-**3** is a previously known compound[14]

^{1}H NMR (500 MHz, $CDCl_3$) δ = 4.77 – 4.63 (m, 2H), 4.35 (dd, $^3J_{H-H}$ =9.5 Hz, $^3J_{H-H}$ = 7.0 Hz, 1H), 2.31 – 2.14 (m, 2H), 1.95 – 1.83 (tt, $^3J_{H-H}$ = 11.8 Hz, $^3J_{H-H}$ = 3.2 Hz, 1H), 1.74 – 1.55 (m, 3H+2H), 1.53 – 1.32 (m, 3H+2H).

^{13}C {^{1}H} NMR (126 MHz, $CDCl_3$) δ = 154.8, 147.4, 110.1, 82.2, 80.5, 39.8, 34.0, 33.0, 26.1, 25.7, 20.6.

MS ESI-TOF+
Calculated [M+Na]$^+$ = 219.0992
Found [M+Na]$^+$ = 219.1017

FT-IR: 1790 cm^{-1} (C=O)

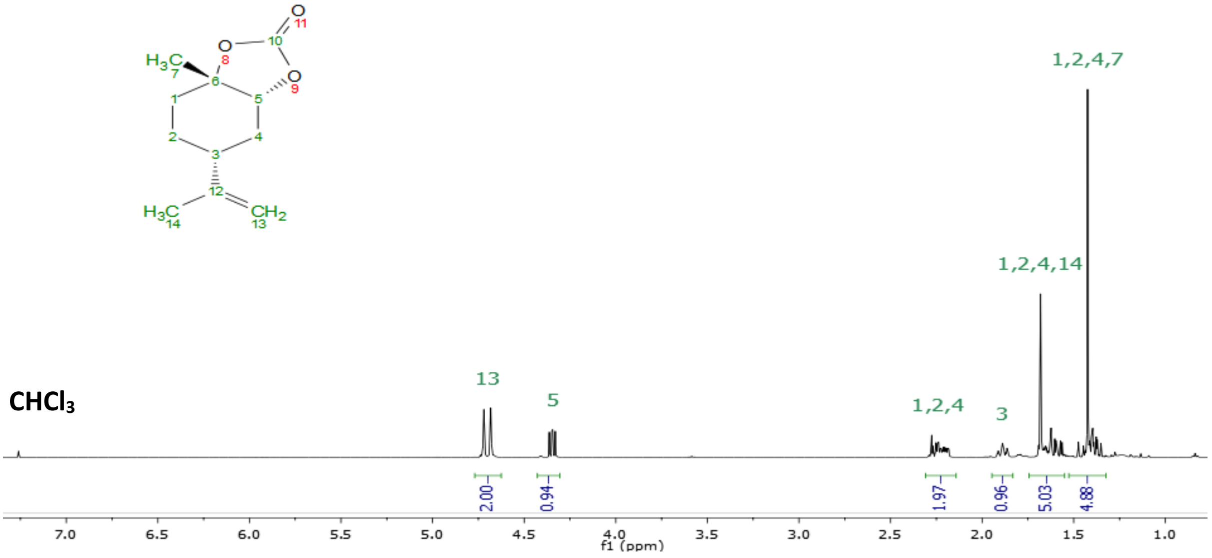

Figure 26 ^{1}H NMR of *trans*-**3**

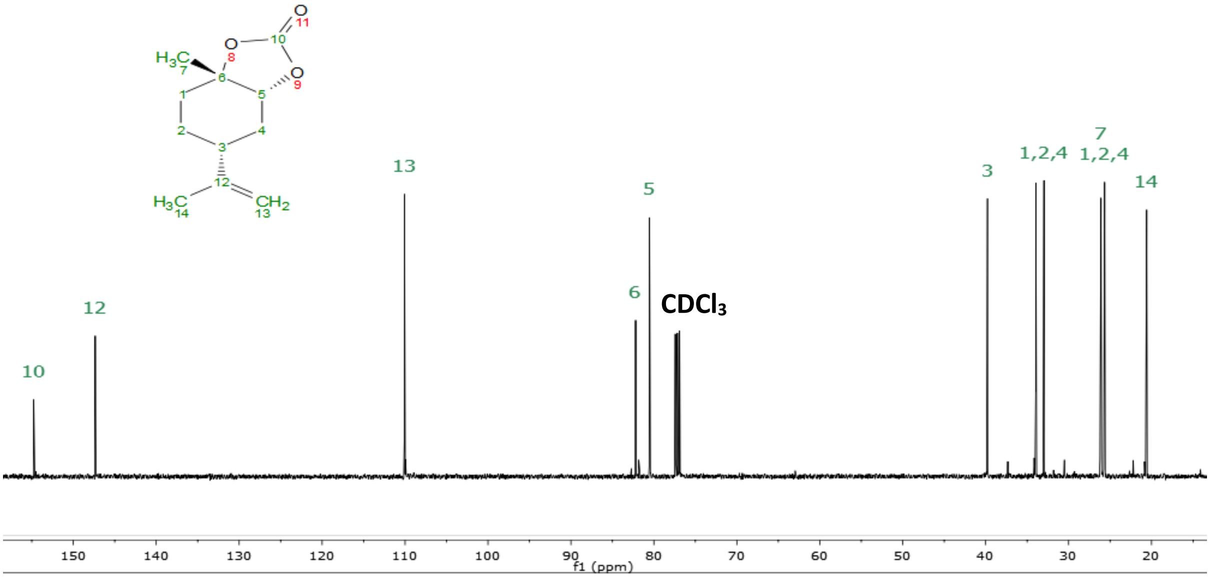

Figure 27 ^{13}C {^{1}H} NMR of *trans*-**3**

Although the presence of *cis*-**3** has been reported by other research grups[14-15] it has not been isolated and fully characterised before. The isomers ratio between *trans*-**3** and *cis*-**3** was calculated using ^{1}H NMR (Figure 24). The ^{1}H NMR spectrum was consistent with previously published data.[15]

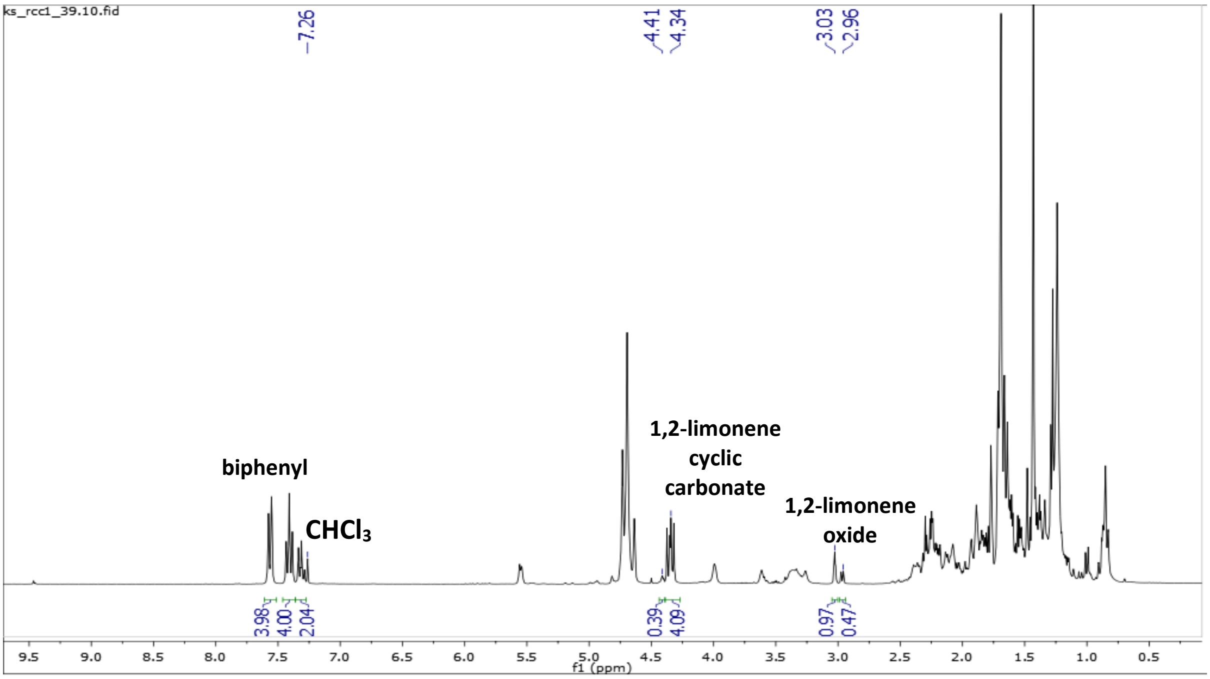

Figure 28 Calculation of isomers ratio in **3**. *Cis* isomer: 4.41 ppm (m), *trans* isomer: 4.34 ppm (dd)

5. 8,9-limonene cyclic carbonate (5)

8,9-limonene cyclic carbonate (5)

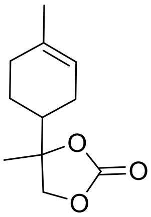

4R,8R-5

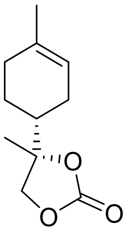

4R,8S-5

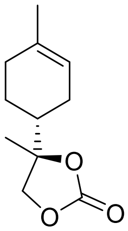

Mixture of isomers

^{1}H NMR (500 MHz, $CDCl_3$) δ = 5.40 – 5.27 (m, 1H), 4.33 – 3.94 (m, 2H), 2.14 – 1.67 (m, 6H), 1.62 (m, 3H), 1.44 ((4R,8R)-**13**) and 1.43 ((4R,8S)-**13**) (s, 3H), 1.30 (m, 1H).

^{13}C NMR (126 MHz, $CDCl_3$) δ = 154.8, 154.8, 134.4, 134.2, 119.0, 118.9, 85.9, 73.1, 72.8, 41.8, 41.7, 30.0, 30.0, 25.7, 25.6, 23.3, 23.0, 23.0, 22.2, 21.9.

TOF MS EI:
Calculated [M] = 196.1099
Found [M] = 196.1102

FT-IR: 1786 cm^{-1} (C=O)

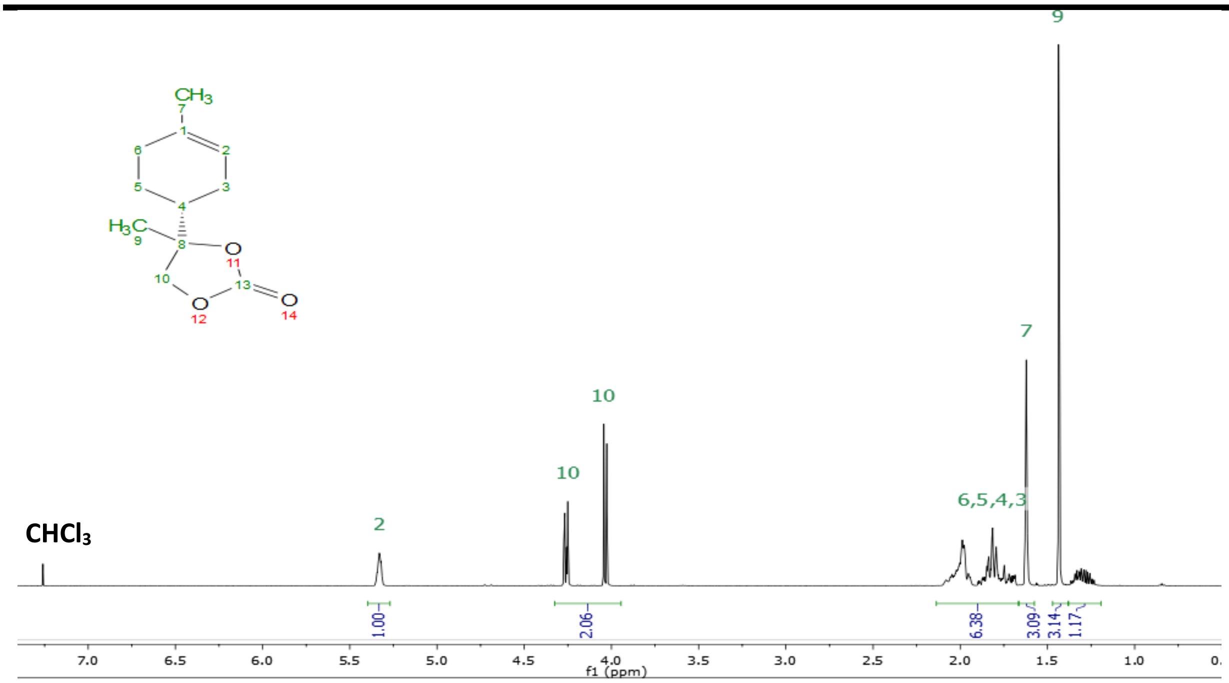

Figure 29 ^{1}H NMR of **5**, mixture of isomers

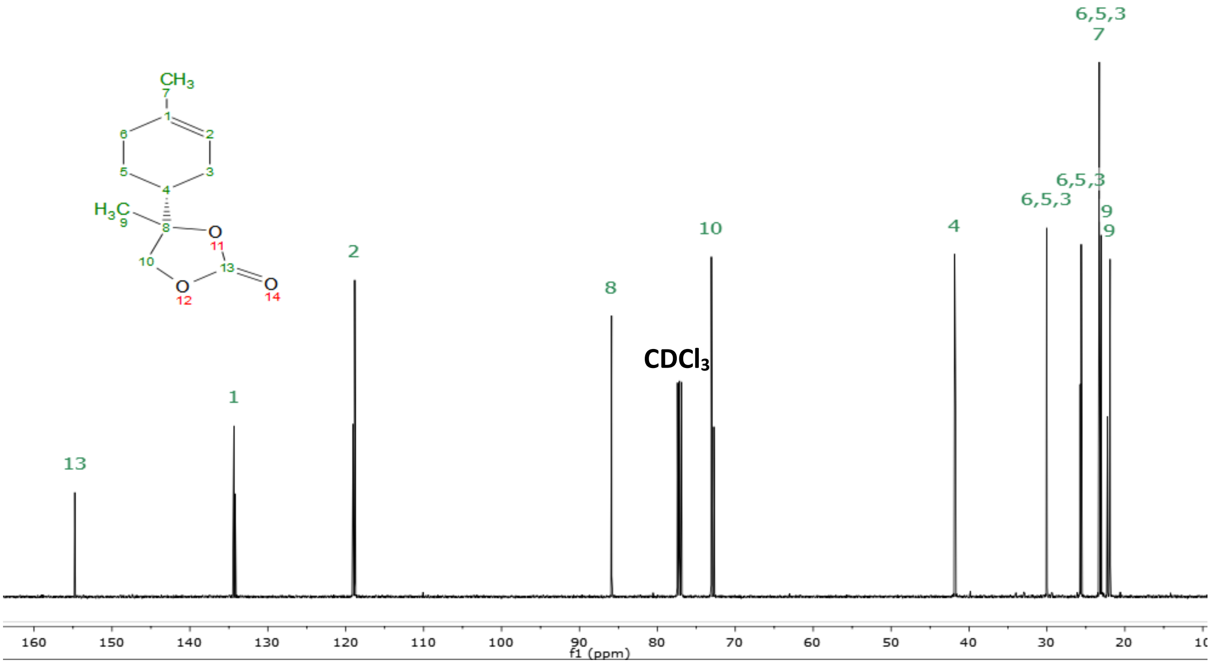

Figure 30 ^{13}C {^{1}H} NMR of **5**, mixture of isomers

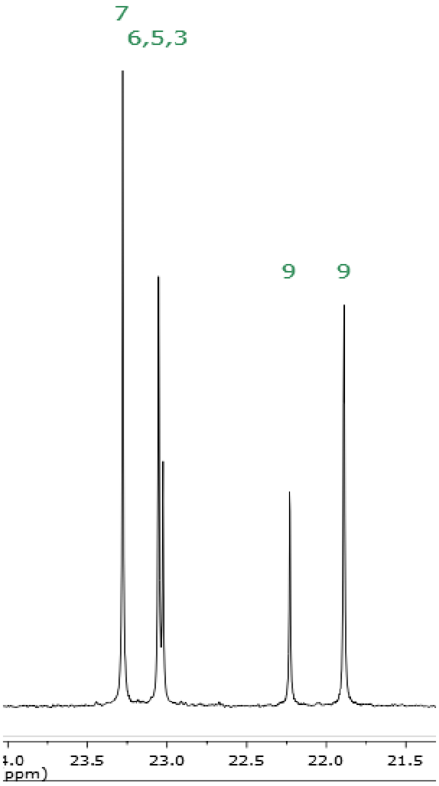

Figure 31 Zoom in on the ^{13}C {^{1}H} NMR of **5** in the range of 23.5-21.5 ppm

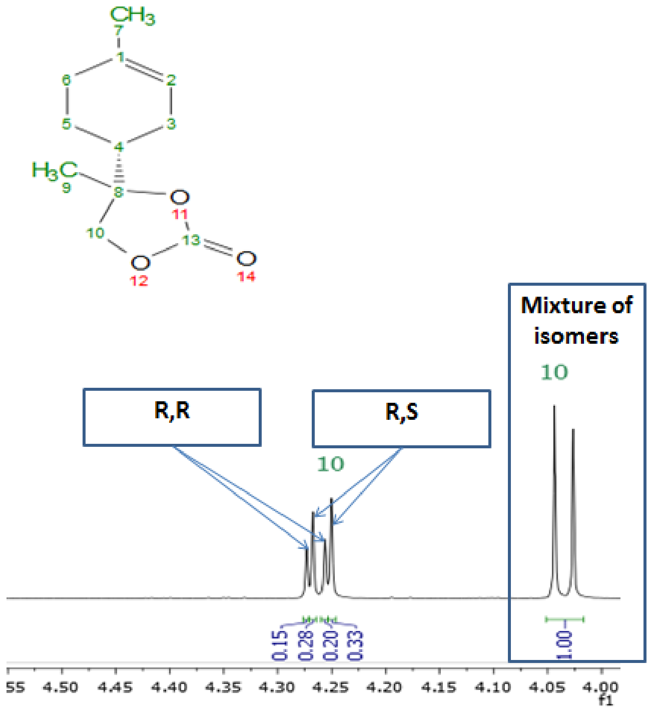

Figure 32 4R, 8R-**5** and 4R, 8S-**5** assignments using ^{1}H NMR and signal from protons at the position 10

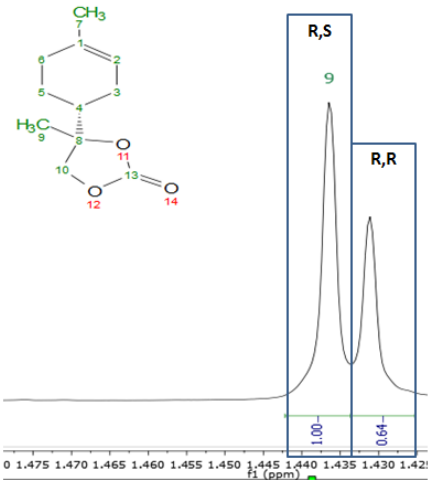

Figure 33 4R, 8R-**5** and 4R, 8S-**5** assignments using ^{1}H NMR and signal from methyl group at the position 9

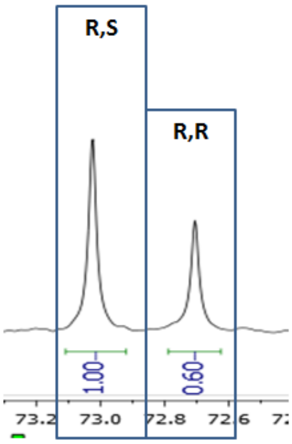

Figure 34 4R, 8R-**5** and 4R, 8S-**5** assignments using inverse gated ^{13}C {^{1}H} NMR and signal from carbon at the position 10

Calculations of **5** stereoisomers [% RR : %RS]			
	^{1}H NMR (H10)	^{1}H NMR (H9)	Inverse gated ^{13}C {^{1}H} NMR (C10)
4R, 8R : 4R, 8S	36:64	39:61	38:62

The isomers assignments are based on the assumption that the carbonation follows the double inversion pathway and the stereoisomers of **4** yield corresponding stereoisomers of **5**.

6. Characterisation of compound 6

Compound **6**

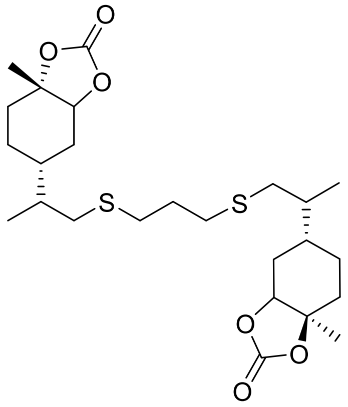

^{1}H NMR (400 MHz, $CDCl_3$) δ = 4.36 – 4.26 (m, 2H), 2.58 – 2.32 (m, 8H), 2.26 – 2.01 (m, 4H), 1.80 (m, 2H), 1.70 – 1.18 (m, 18H), 0.92 (2xd, *J*=6.9, 6H).

^{13}C NMR (101 MHz, $CDCl_3$) δ = 154.7, 82.5, 80.8, 80.7, 37.3, 37.2, 37.1, 35.7, 35.7, 32.9, 32.8, 32.7, 31.7, 31.5, 31.5, 30.3, 29.4, 29.3, 28.6, 26.0, 26.0, 24.5, 24.4, 23.4, 21.9, 15.9, 15.8.

TOF MS ASAP:
Calculated $[M+H]^+$ = 501.2345
Found $[M+H]^+$ = 501.2343

FT-IR: 1792 cm^{-1} (C=O)

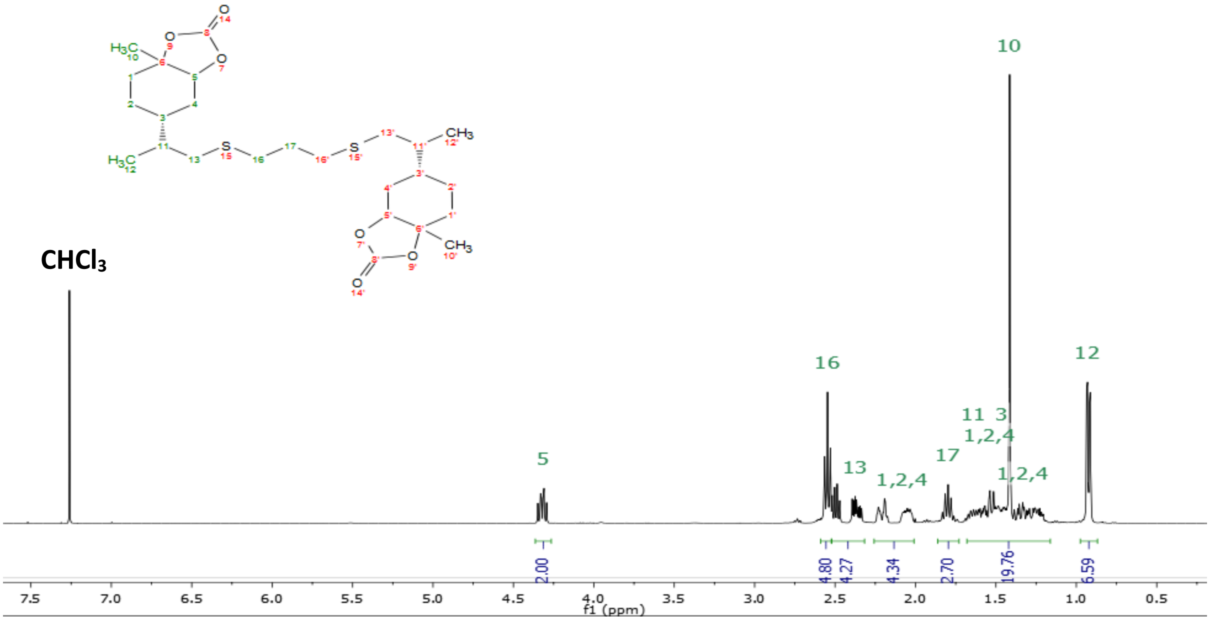

Figure 35 ^{1}H NMR of **6**

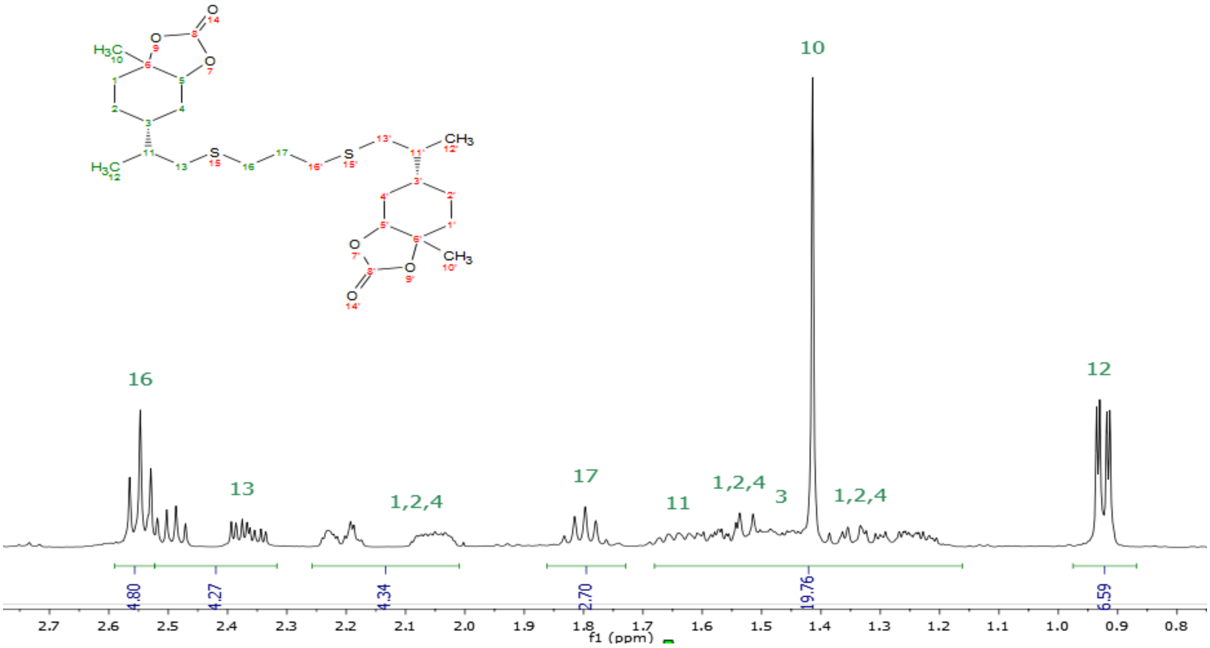

Figure 36 Zoom in on ^{1}H NMR spectrum of **6** in the range of 2.7 ppm – 0.8 ppm

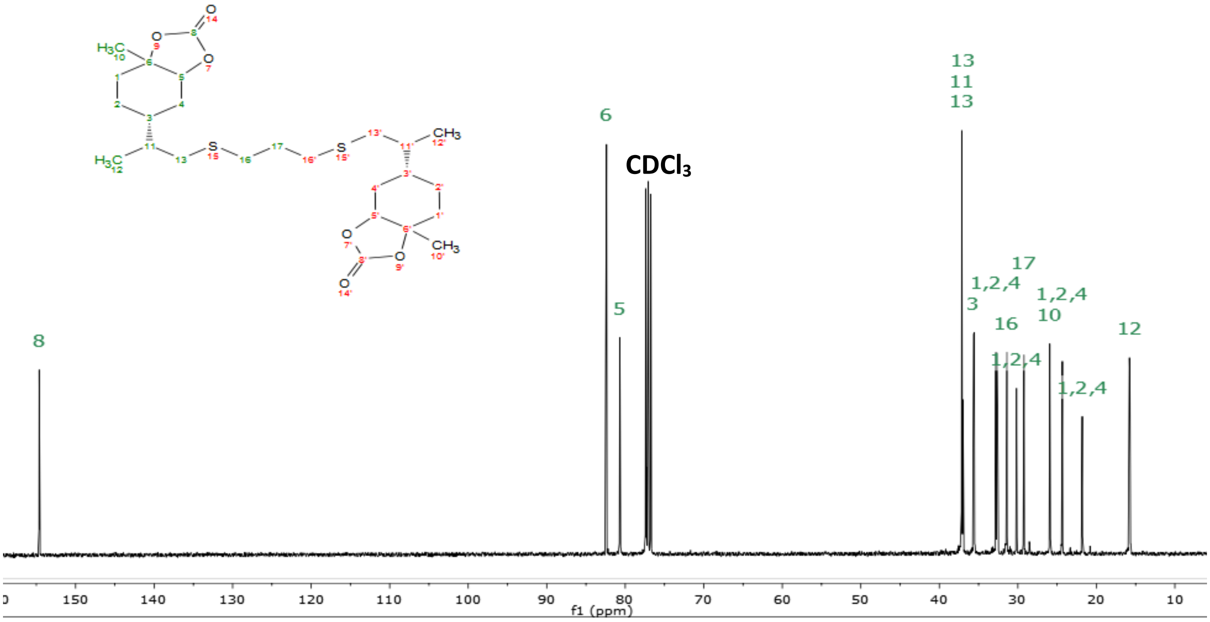

Figure 37 ^{13}C {^{1}H} NMR of **6**

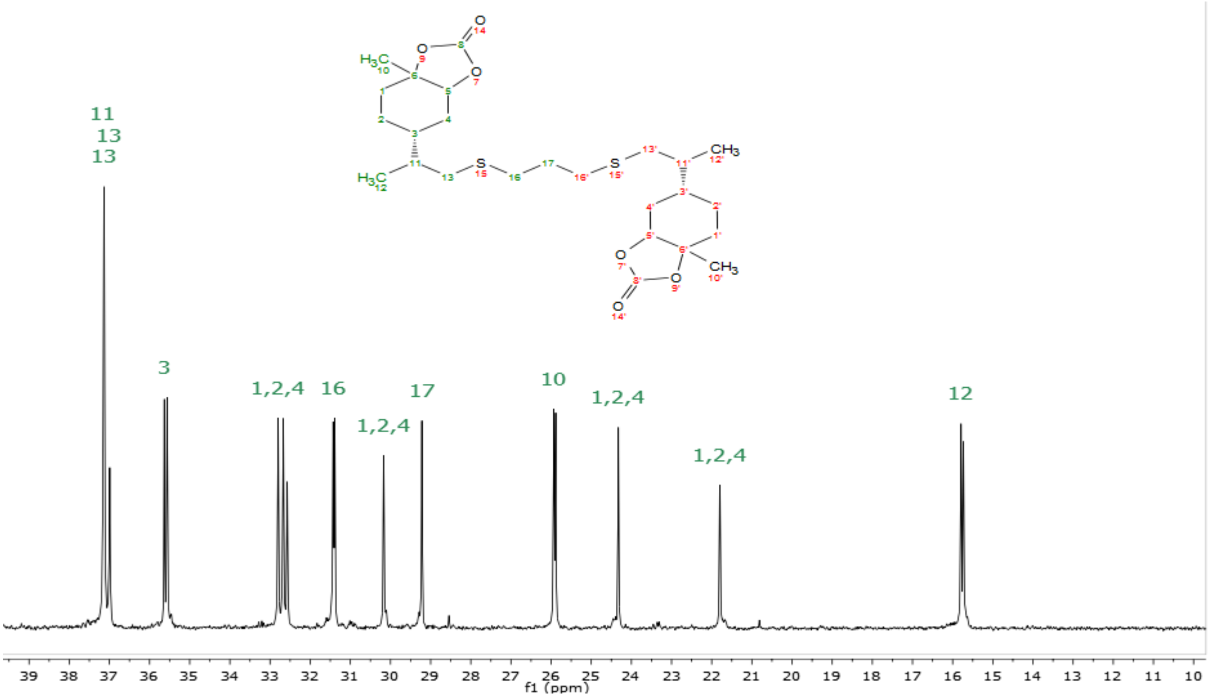

Figure 38 Zoom in on ^{13}C {^{1}H} NMR spectrum of **6** in the range 39 ppm – 10 ppm

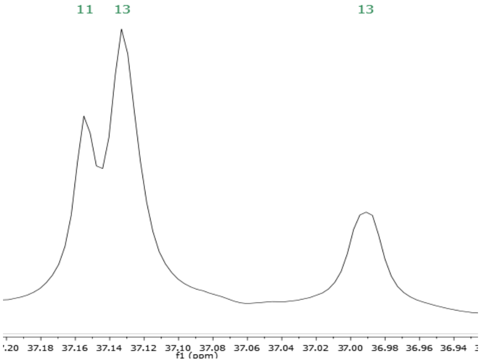

Figure 39 Zoom in on ^{13}C {^{1}H} NMR spectrum of **6** in the region 37.2 ppm – 36.9 ppm

7. Characterisation of compound 7

Compound **7**

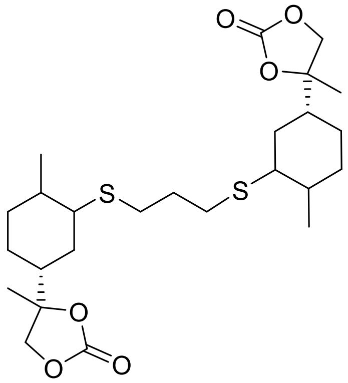

^{1}H NMR(400 MHz, $CDCl_3$): δ = 4.36 – 4.19 (m, 2H), 4.02 (m, 2H), 3.03 – 2.51 (m, 6H), 2.14 – 2.03 (m, 2H), 1.98 – 0.90 (m, 28H) ppm

^{13}C NMR (126 MHz, $CDCl_3$) δ = 154.71, 85.71, 73.65, 73.16, 50.53, 50.04, 49.85, 45.91, 39.75, 37.44, 36.52, 34.71, 33.18, 31.53, 29.06, 26.15, 22.31, 21.71, 20.65, 20.27, 19.56, 18.63, 12.26.

TOF MS ASAP:

Calculated $[M+H]^+$ = 501.2345

Found $[M+H]^+$ = 501.2344

FT-IR: 1788 cm^{-1} (C=O)

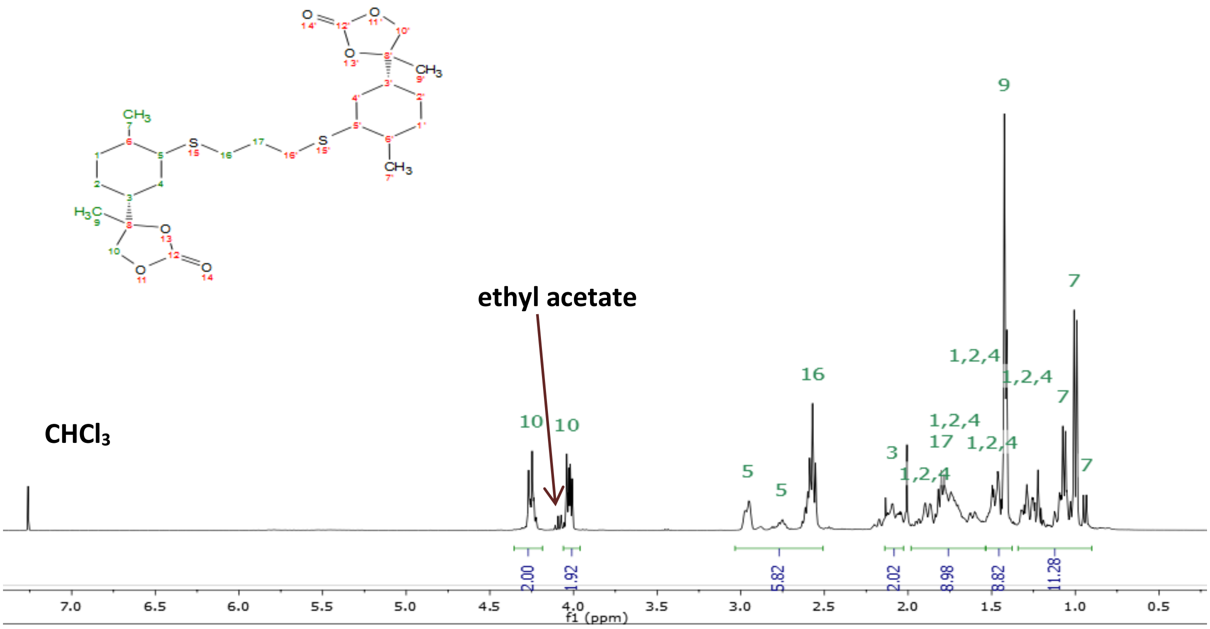

Figure 40 ^{1}H NMR of **7**

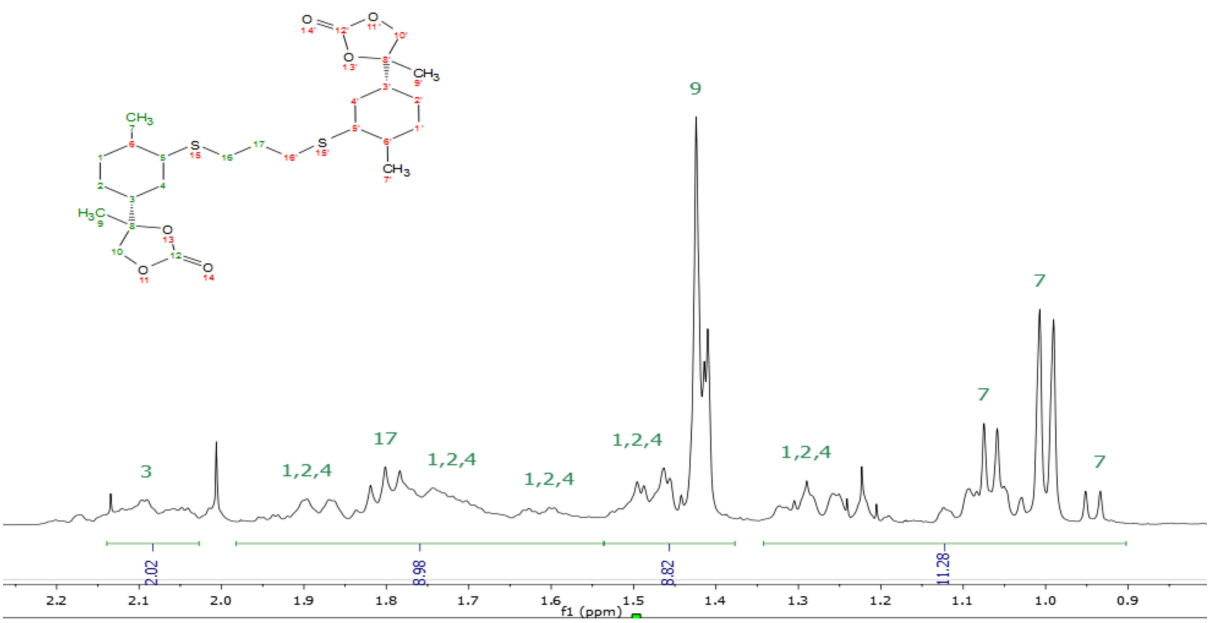

Figure 41 Zoom in on ^{1}H NMR spectrum in the range of 2.2 ppm – 0.9 ppm

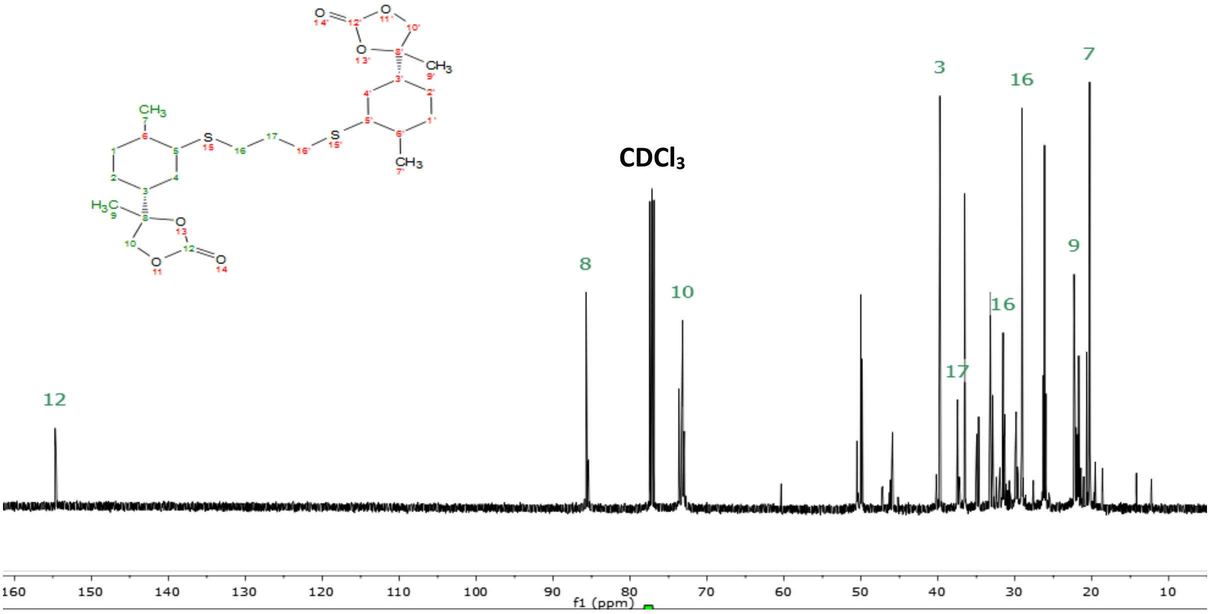

Figure 42 ^{13}C {^{1}H} NMR of **7**

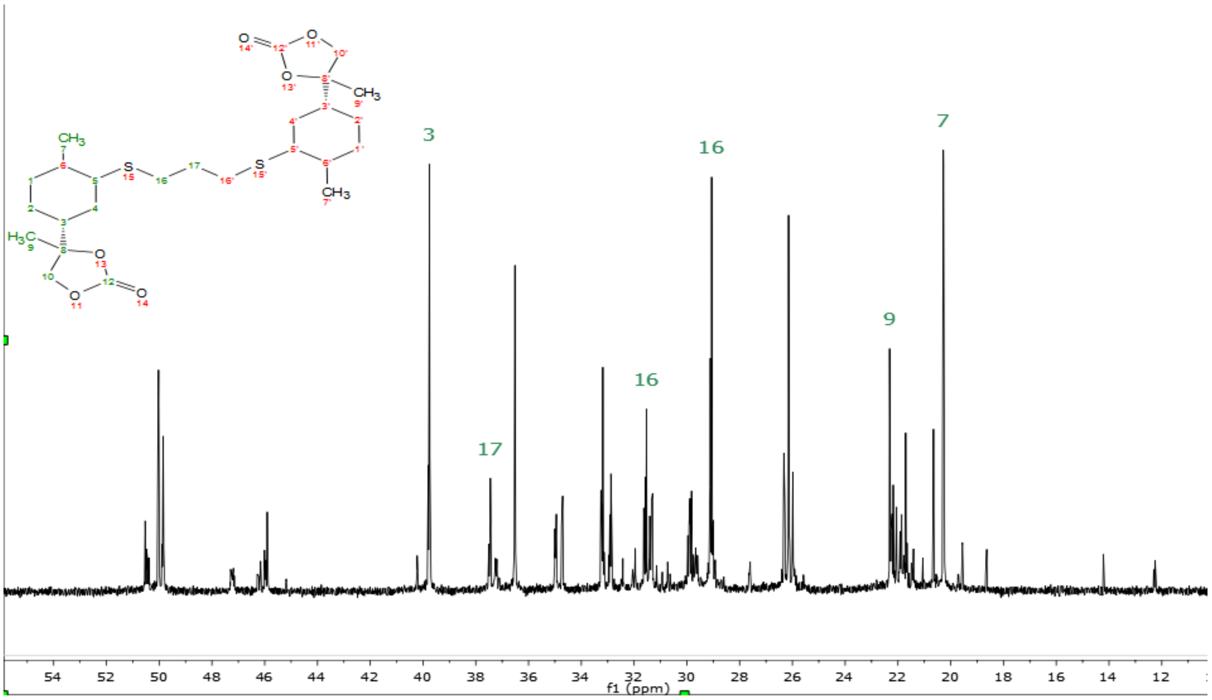

Figure 43 Zoom in on spectrum in the range of 54 ppm - 11.5 ppm

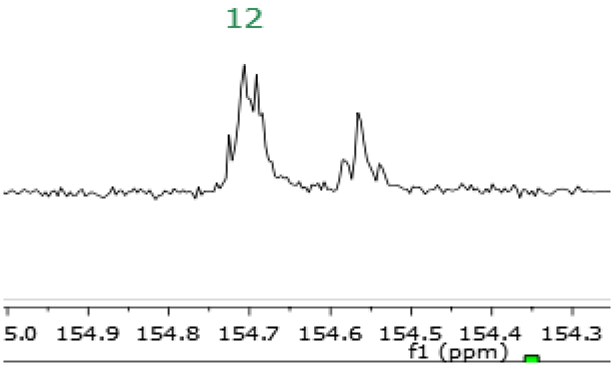

Figure 44 Zoom in on ^{13}C {^{1}H} NMR spectrum in the carbonyl region. The complexity of C=O signal indicates presence of isomers mixture

8. 8,9-limonene aldehyde (41)

Compound **41**

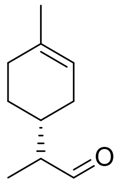

^{1}H NMR (400 MHz, $CDCl_3$) δ = 9.65 (2xd, $^3J_{H-H}$ = 2.5 Hz, 1H), 5.35 (m, 1H), 2.26 (m, 1H), 2.13 - 1.54 (m, 5H+3H), 1.35 (m, 2H), 1.06 (2xd, $^3J_{H-H}$ =7.0 Hz, 3H).

^{13}C {1H} NMR (101 MHz, $CDCl_3$) δ = 205.5, 205.4, 134.2, 134.2, 120.1, 120.0, 51.1, 50.8, 34.5, 34.4, 30.8, 30.3, 30.1, 29.8, 28.2, 27.4, 25.6, 23.6, 23.5, 10.5, 10.4.

ESI QTOF MS:
Calculated $[M-H]^-$ = 151.1117
Found $[M-H]^-$ = 151.1134

FT-IR: 1707 cm^{-1} (C=O)

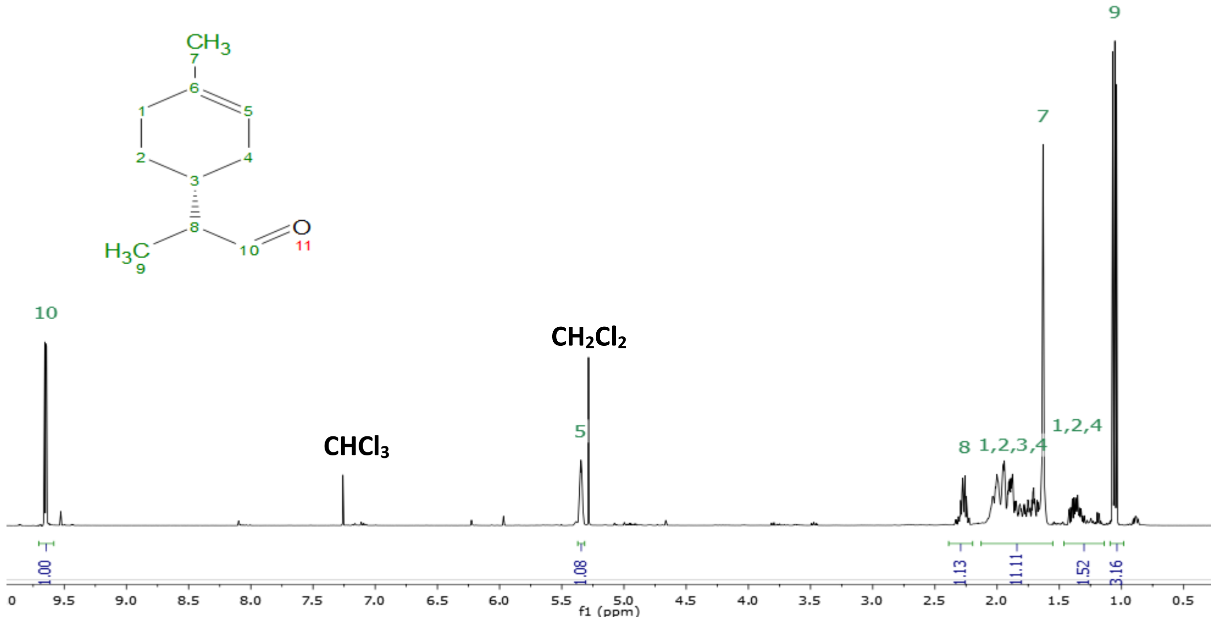

Figure 45 ^{1}H NMR of **41**

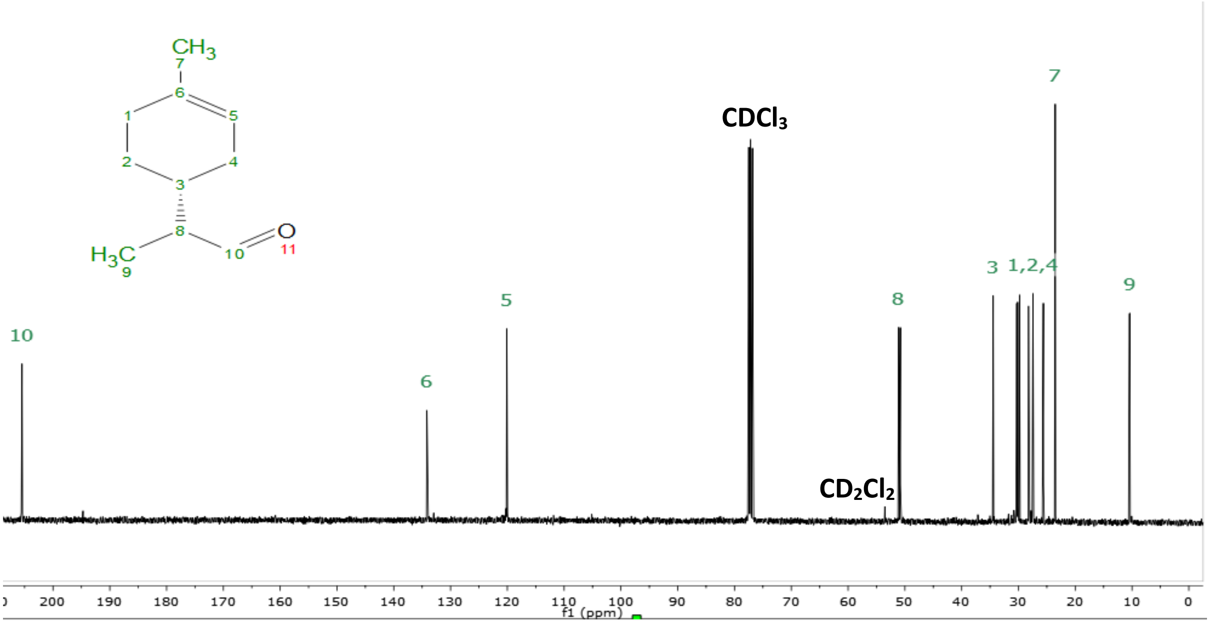

Figure 46 ^{13}C {^{1}H} NMR of **41**

XI PMI calculations

Calculations for PMI presented in Table 8, PMI is defined as the ratio of mass of all materials used in the synthesis to the mass of recovered limonene carbonate.

Table 8 Entry 1

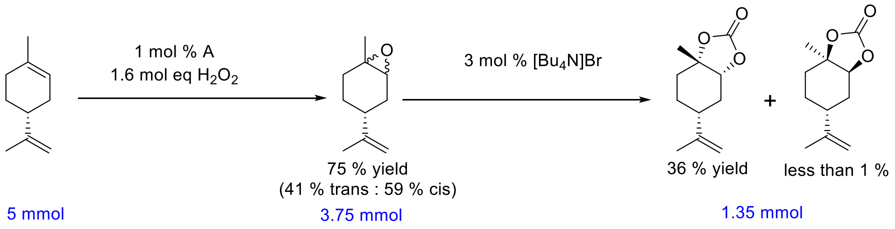

Oxidation: mass of materials used for 5 mmol limonene = 1.7 g (This work, see Figure 8 in the main paper)

**Mass of materials:**	
limonene	$5\,mmol \cdot 136\frac{mg}{mmol} = 680\,mg = 0.680\,g$
H_2O_2	$8\,mmol \cdot 34\frac{mg}{mmol} = 272\,mg = 0.272\,g$<br>$H_2O_2$ was used as 30 % aqueous solution:<br>$30\,\% - 0.272\,g$<br>$100\,\% - X$<br>$X = \frac{100\,\% \cdot 0.272\,g}{30\,\%} = 0.907\,g$
1 mol % Venturello	$0.01 \cdot 5\,mmol \cdot 2255\frac{mg}{mmol} = 113\,mg = 0.113\,g$
overall	1.7 g used for 5 mmol limonene oxidation
Mass of recovered product<br>75 % yield (41 % trans: 59 % cis)	$0.75 \cdot 5\,mmol \cdot 152\frac{mg}{mmol} = 570\,mg = 0.570\,g$

Carbonation: mass of materials used for 3.75 mmol limonene oxide = 3.48 g

**Mass of materials:**	
Limonene oxide	$2.6\ mmol \cdot 152 \frac{mg}{mmol} = 395\ mg = 0.395\ g$
3 mol % Bu_4NBr	$0.03 \cdot 2.6\ mmol \cdot 322 \frac{mg}{mmol} = 25\ mg = 0.025\ g$
CO_2	75 bar at 40 °C ; CO_2 density = 0.238 g/mL<br>10 mL reactor<br>2.38 g CO_2
overall	Not including limonene oxide – this mass has been included in oxidation reactions<br>2.41 g per 2.6 mmol lim ox
Mass of recovered product from limonene epoxidised with Venturello catalyst<br>36 % yield	$0.36 \cdot 3.75\ mmol \cdot 196 \frac{mg}{mmol} = 265\ mg = 0.265\ g$

$$\boldsymbol{PMI = \frac{1.7\ g + 3.48\ g}{0.265\ g} = 19.5}$$

Table 8 Entry 2

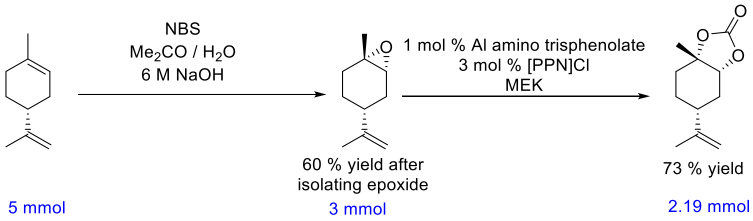

Oxidation: mass of materials used for 5 mmol limonene = 10.9 g (according to Green Chem., 2016, 18, 760-770)

**Mass of materials:**	
limonene	$8.8\ mol \cdot 136 \frac{g}{mol} = 1197\ g$
NBS	$9.2\ mol \cdot 178 \frac{g}{mol} = 1638\ g$
acetone	$4.5\ L \cdot 780 \frac{g}{L} = 3510\ g$
Water	$(1\ L + 1.5\ L + 1.5\ L) \cdot 1000 \frac{g}{L} = 4500\ g$
Diethyl ether	$(3\ L + 3\ L) \cdot 710 \frac{g}{L} = 4260\ g$
Bicarbonate solution	$1.5\ L \cdot 1100 \frac{g}{L} = 1650\ g$
6 M NaOH	$2\ L \cdot 1220 \frac{g}{L} = 2440\ g$
overall	19 195 g used for 8.8 mol limonene
Mass of recovered product (assuming 100 % recovery from distillation), crude reaction mixture: 1.4 L<br>Density of crude reaction mixture assumed the same as limonene density 0.930 g/cm^3, Yield 60 %	$recovered\ product = 0.6 \cdot \left(1.4 \cdot 930 \frac{g}{L}\right) = 781\ g\ (5.1\ mol)$

Carbonation: mass of materials used for 3 mmol limonene oxide = 0.900 g (according to ChemSusChem, 2016, 9, 1304-1311)

**Mass of materials:**	
Limonene oxide	$5\ mmol \cdot 152 \frac{mg}{mmol} = 760\ mg = 0.760\ g$
1 mol % Al complex	$0.01 \cdot 5\ mmol \cdot 700 \frac{mg}{mmol} = 35\ mg = 0.035\ g$
3 mol % PPNCl	$0.03 \cdot 5\ mmol \cdot 573 \frac{mg}{mmol} = 86\ mg$
MEK	$1\ mL \cdot 0.805 \frac{g}{mL} = 0.805\ g$
CO_2	10 bar at 20 ^oC ; CO_2 density = 0.0191 g/mL<br>30 mL reactor<br>0.573 g CO_2
overall	Not including limonene oxide – this mass has been included in oxidation reactions<br>1.499 g per 5 mmol limonene oxide
Mass of recovered product from *trans*-1,2-limonene oxide	$0.73 \cdot 5\ mmol \cdot 196 \frac{mg}{mmol} = 715\ mg = 0.715\ g$

$$\boldsymbol{PMI = \frac{10.9\ g + 0.9\ g}{0.429\ g} = 27.5}$$

Table 8 Entry 3

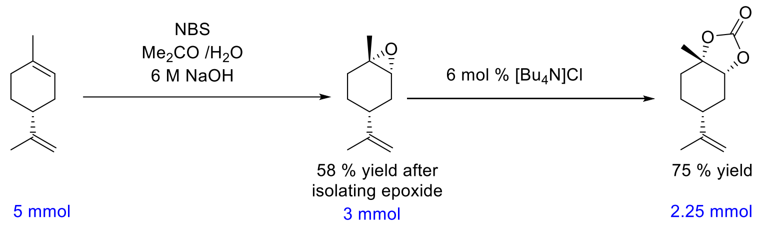

Oxidation: mass of materials used for 5 mmol limonene = 10.9 g (according to Green Chem., 2016, 18, 760-770)

The same as previous entry (Table 8 Entry 2)

Carbonation: mass of materials used for 3 mmol limonene oxide = 1.07 g (according to J. CO_2 Util., 2019, 29, 126-133)

**Mass of materials:**	
Limonene oxide	$44\ mmol \cdot 152 \frac{mg}{mmol} = 6688\ mg = 6.688\ g$
6 mol % Bu_4NCl	$0.06 \cdot 44\ mmol \cdot 110 \frac{mg}{mmol} = 290\ mg = 0.290\ g$
DMF	$10\ mL \cdot 0.944 \frac{g}{mL} = 9.44\ g$
CO_2	40 bar at 120 ºC ; CO_2 density = 0.0589 g/mL<br>100 mL reactor<br>5.89 g CO_2
overall	Not including limonene oxide – this mass has been included in oxidation reactions<br>15.62 g per 44 mmol limonene oxide
Mass of recovered product from *trans*-1,2-limonene oxide	$0.75 \cdot 44\ mmol \cdot 196 \frac{mg}{mmol} = 6468\ mg = 6.468\ g$

$$PMI = \frac{10.9\ g + 1.07\ g}{0.441\ g} = 27.1$$

Table 8 Entry 4

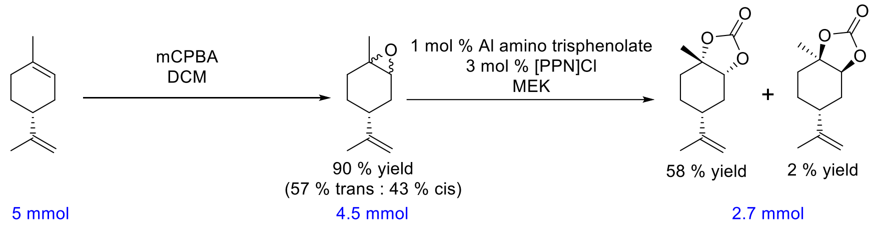

Oxidation: mass of materials used for 5 mmol limonene = 25 g

All reported literature use commercially available 1,2-limonene oxide, as no other data is available, it is assumed that commercially available 1,2-limonene oxide is synthesised via standard mCPBA oxidation. The dilution was set similar to that used in NBS oxidation (although mCPBA oxidations on laboratory scale usually use higher dilution).

Yields of this epoxidation were not available, yield was assumed as 90 %.

Mass of materials:	
limonene	$$5\ mmol\ \cdot 136\frac{mg}{mmol} =\ 680\ mg = 0.680\ g$$
mCPBA	$$5.5\ mmol\ \cdot 173\frac{mg}{mmol} = 952\ mg = 0.952\ g$$<br>mCPBA is sold as solid containing only 70 % mCPBA<br>$70\ \% - 0.952\ g$<br>$100\ \% - X$<br>$X = \frac{100\ \%\ \cdot 0.952\ g}{70\ \%} = 1.36\ g$
DCM	$$5\ mL\ \cdot 1.33\frac{g}{mL} = 6.65\ g$$
Bicarbonate solution	$$5\ mL\ \cdot 1.1\frac{g}{mL} = 5.5\ g$$
Brine solution	$$5\ mL\ \cdot 1.1\frac{g}{mL} = 5.5\ g$$
water	$$5\ mL\ \cdot 1\frac{g}{mL} = 5\ g$$
overall	25 g used for 5 mmol limonene oxidation
Mass of recovered product (assuming 100 % recovery from distillation)<br>No data available on mCPBA oxidation of limonene, 90 % yield was assumed	$$0.9 \cdot 5\ mmol \cdot 152\frac{mg}{mmol} = 684\ mg = 0.684\ g$$

Carbonation: mass of materials used for 4.5 mmol limonene oxide = 1.349 g (according to ChemSusChem, 2016, 9, 1304-1311)

Mass of materials:	
Limonene oxide	$$5\ mmol\ \cdot 152\frac{mg}{mmol} =\ 760\ mg = 0.760\ g$$
1 mol % Al complex	$$0.01\ \cdot 5\ mmol \cdot 700\frac{mg}{mmol} = 35\ mg = 0.035\ g$$
3 mol % PPNCl	$$0.03\ \cdot 5\ mmol\ \cdot 573\frac{mg}{mmol} = 86\ mg$$
MEK	$$1\ mL\ \cdot 0.805\frac{g}{mL} = 0.805\ g$$
CO_2	10 bar at 20 ^oC ; CO_2 density = 0.0191 g/mL<br>30 mL reactor<br>0.573 g CO_2
overall	Not including limonene oxide – this mass has been included in oxidation reactions<br>1.499 g per 5 mmol limonene oxide
Mass of recovered product from commercially available 1,2-limonene oxide	$$0.6 \cdot 5\ mmol\ \cdot 196\frac{mg}{mmol} = 588\ mg = 0.588\ g$$

$$PMI = \frac{25\ g + 1.349\ g}{0.529\ g} = 50$$

Table 8 Entry 5

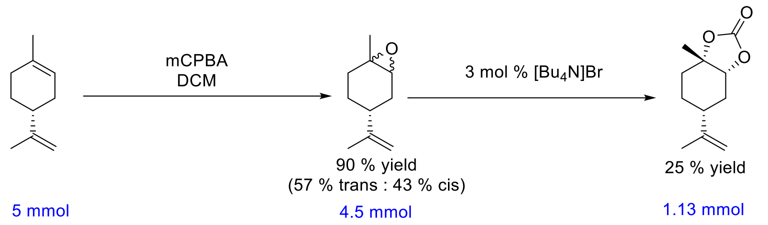

Oxidation: mass of materials used for 5 mmol limonene = 25 g

The same as Table 8 Entry 4

Carbonation: mass of materials used for 4.5 mmol limonene oxide = 4.171 g (This work, see Table 3 Entry 2 in the main paper)

**Mass of materials:**	
Limonene oxide	$2.6\ mmol \cdot 152 \frac{mg}{mmol} = 395\ mg = 0.395\ g$
3 mol % Bu_4NBr	$0.03 \cdot 2.6\ mmol \cdot 322 \frac{mg}{mmol} = 25\ mg = 0.025\ g$
CO_2	75 bar at 40 ^oC ; CO_2 density = 0.238 g/mL<br>10 mL reactor<br>2.38 g CO_2
overall	Not including limonene oxide – this mass has been included in oxidation reactions<br>2.41 g per 2.6 mmol lim ox
Mass of recovered product from commercially available 1,2-limonene oxide<br>25 % yield	$0.25 \cdot 2.6\ mmol \cdot 196 \frac{mg}{mmol} = 127\ mg = 0.127\ g$

$$PMI = \frac{25\ g + 4.171\ g}{0.221\ g} = 132$$

XII References

1. Hintermair, U.; Roosen, C.; Kaever, M.; Kronenberg, H.; Thelen, R.; Aey, S.; Leitner, W.; Greiner, L., A Versatile Lab to Pilot Scale Continuous Reaction System for Supercritical Fluid Processing. *Org. Process Res. Dev.* **2011,** *15* (6), 1275-1280.
2. Venturello, C.; Daloisio, R., Quaternary Ammonium Tetrakis(Diperoxotungsto)Phosphates(3-) as a New Class of Catalysts for Efficient Alkene Epoxidation with Hydrogen-Peroxide. *J. Org. Chem.* **1988,** *53* (7), 1553-1557.
3. Cunningham, W. B.; Tibbetts, J. D.; Hutchby, M.; Smug, K. A.; Davidson, M. G.; Hintermair, U.; Plucinski, P.; Bull, S. D., Sustainable Catalytic Protocols for the Solvent Free Epoxidation and (anti)-Dihydroxylation of the Alkene Bonds of Biorenewable Terpene Feedstocks Using H2O2 as Oxidant. *under review*.
4. Steiner, D.; Ivison, L.; Goralski, C. T.; Appell, R. B.; Gojkovic, J. R.; Singaram, B., A facile and efficient method for the kinetic separation of commercially available cis- and trans-limonene epoxide. *Tetrahedron-Asymmetr* **2002,** *13* (21), 2359-2363.
5. Kumar, S. C. S.; Manjunatha, J. R.; Srinivas, P.; Bettadaiah, B. K., Eco-friendly kinetic separation of trans-limonene and carvomenthene oxides. *J. Chem. Sci. (Bangalore, India)* **2014,** *126* (3), 875-880.
6. Hauenstein, O.; Reiter, M.; Agarwal, S.; Rieger, B.; Greiner, A., Bio-based polycarbonate from limonene oxide and CO2 with high molecular weight, excellent thermal resistance, hardness and transparency. *Green Chem.* **2016,** *18* (3), 760-770.
7. Domaille, P. J., Vanadium(V) Substituted Dodecatungstophosphates. In *Inorg. Synth.*, Hervéa, G.; Téazéa, A., Eds. John Wiley & Sons, Inc.: 1990; Vol. 27, pp 96-104.
8. Knoth, W. H.; Harlow, R. L., New Tungstophosphates - Cs6w5p2o23, Cs7w10po36, and Cs7na2w10po37. *J. Am. Chem. Soc.* **1981,** *103* (7), 1865-1867.
9. Domaille, P. J.; Harlow, R. L., Synthesis and structural characterization of the first phosphorus-centered Baker-Figgis .gamma.-dodecametalate: .gamma.-Cs5[PV2W10O40].cntdot.xH2O. *J. Am. Chem. Soc.* **1986,** *108* (8), 2108-2109.
10. Kamata, K.; Sugahara, K.; Yonehara, K.; Ishimoto, R.; Mizuno, N., Efficient Epoxidation of Electron-Deficient Alkenes with Hydrogen Peroxide Catalyzed by [γ-PW10O38V2(μ-OH)2]3−. *Chemistry – A European Journal* **2011,** *17* (27), 7549-7559.
11. Carman, R. M.; Devoss, J. J.; Greenfield, K. L., The Diastereomeric 8,9-Epoxides of Limonene (the 8,9-Epoxy-p-menth-1-enes). *Aust. J. Chem.* **1986,** *39* (3), 441.
12. Firdaus, M.; Montero de Espinosa, L.; Meier, M. A. R., Terpene-Based Renewable Monomers and Polymers via Thiol–Ene Additions. *Macromolecules* **2011,** *44* (18), 7253-7262.
13. Byrne, C. M.; Allen, S. D.; Lobkovsky, E. B.; Coates, G. W., Alternating Copolymerization of Limonene Oxide and Carbon Dioxide. *J. Am. Chem. Soc.* **2004,** *126* (37), 11404-11405.
14. Fiorani, G.; Stuck, M.; Martín, C.; Belmonte, M. M.; Martin, E.; Escudero-Adán, E. C.; Kleij, A. W., Catalytic Coupling of Carbon Dioxide with Terpene Scaffolds: Access to Challenging Bio-Based Organic Carbonates. *ChemSusChem* **2016,** *9* (11), 1304-1311.
15. Longwitz, L.; Steinbauer, J.; Spannenberg, A.; Werner, T., Calcium-Based Catalytic System for the Synthesis of Bio-Derived Cyclic Carbonates under Mild Conditions. *ACS Catalysis* **2018,** *8* (1), 665-672.
